# Redox Regulation of PPAR*γ* in Polarized Macrophages

**DOI:** 10.1155/2020/8253831

**Published:** 2020-07-01

**Authors:** Verena Trümper, Ilka Wittig, Juliana Heidler, Florian Richter, Bernhard Brüne, Andreas von Knethen

**Affiliations:** ^1^Institute of Biochemistry I, Goethe-University Frankfurt, 60590 Frankfurt am Main, Germany; ^2^Functional Proteomics, SFB 815 Core Unit, Goethe-University Frankfurt, 60590 Frankfurt am Main, Germany; ^3^Institute of Pharmaceutical and Biochemical Science, Johannes Gutenberg-University Mainz, 55099 Mainz, Germany; ^4^Branch for Translational Medicine and Pharmacology, Fraunhofer Institute for Molecular Biology and Applied Ecology IME, Theodor-Stern-Kai 7, 60596 Frankfurt/Main, Germany; ^5^Department of Anaesthesiology, Intensive Care Medicine and Pain Therapy, University Hospital Frankfurt, 60590 Frankfurt, Germany

## Abstract

The peroxisome proliferator-activated receptor (PPAR*γ*) is a central mediator of cellular lipid metabolism and immune cell responses during inflammation. This is facilitated by its role as a transcription factor as well as a DNA-independent protein interaction partner. We addressed how the cellular redox milieu in the cytosol and the nucleus of lipopolysaccharide (LPS)/interferon-*γ*- (IFN*γ*-) and interleukin-4- (IL4-) polarized macrophages (M*Φ*) initiates posttranslational modifications of PPAR*γ*, that in turn alter its protein function. Using the redox-sensitive GFP2 (roGFP2), we validated oxidizing and reducing conditions following classical and alternative activation of M*Φ*, while the redox status of PPAR*γ* was determined via mass spectrometry. Cysteine residues located in the zinc finger regions (amino acid fragments AA 90-115, AA 116-130, and AA 160-167) of PPAR*γ* were highly oxidized, accompanied by phosphorylation of serine 82 in response to LPS/IFN*γ*, whereas IL4-stimulation provoked minor serine 82 phosphorylation and less cysteine oxidation, favoring a reductive milieu. Mutating these cysteines to alanine to mimic a redox modification decreased PPAR*γ*-dependent reporter gene transactivation supporting a functional shift of PPAR*γ* associated with the M*Φ* phenotype. These data suggest distinct mechanisms for regulating PPAR*γ* function based on the redox state of M*Φ*.

## 1. Introduction

Reactive oxygen species (ROS) fulfill important mediator functions in a variety of cellular signaling pathways regulating aspects of inflammation, cell proliferation and differentiation, apoptosis, iron homeostasis, and defense mechanisms [1–3]. Furthermore, ROS trigger redox-based posttranslational modifications (PTMs) changing protein folding and stability. The efficiency of these chemical reactions depends on different criteria including rate constants between oxidants and amino acids, residue accessibility, its pKa, and, additionally, the properties of neighboring amino acids [4]. One of the most important targets of redox-based modifications is redox-sensitive thiols of cysteines [5]. Cysteines are often localized in the functional core units of protein domains. Thus, their thiols shape protein conformation by forming disulfide bridges with other cysteines or tying metal ions such as zinc. Furthermore, nucleophilic thiolates can be catalytically active, and the connection of the cysteine residues with other functional residues ensures special properties of proteins. Consequently, alterations of their chemical behavior drastically change the function of the whole protein. There are many proteins, containing more than one functional protein domain including differently acting cysteines. One example is the peroxisome proliferator-activated receptor gamma (PPAR*γ*).

PPAR*γ* is a ubiquitously expressed, ligand-dependent transcription factor. It is a major player in regulating lipid metabolism of cells and controlling immune cell responses [6–8]. As a member of the PPAR superfamily, the protein has a typical protein domain structure. Starting from the N-terminus, PPARs contain a ligand-independent transactivator domain (AF1), a DNA-binding domain (DBD), a hinge domain (HD), and a dimerization, respectively, ligand-binding domain (LBD) with a ligand-dependent transactivator function (AF2) [9, 10]. This domain structure mediates DNA-binding as well as protein-protein interactions of PPAR*γ*. Moreover, the AF-domains contribute to binding efficiencies of coactivators and -repressors following ligand binding. PPAR*γ* contains ten cysteine residues. Eight of them are located in the DBD forming zinc finger motifs, one is positioned in the HD with unknown function, and one resides in the LBD, involved in ligand binding [11]. Considering the structure of PPAR*γ*, it regulates gene transcription when bound to DNA or by scavenging transcription factors, corepressors or coactivators, from binding to target genes by protein-protein interactions. Specifically, it forms a DNA-binding heterodimer together with retinoid X receptor (RXR) and recruits different coactivators to initiate transcription [12]. Furthermore, PPAR*γ* directly interacts with DNA-bound corepressors causing their stabilization and transrepression of transcription [8]. Based on these different modes of action, PPAR*γ* affects various cellular responses also during inflammation.

Macrophages (M*Φ*), one of the first immune cells starting inflammatory and innate immune responses, express PPAR*γ*. Recognizing pathogen-associated molecular patterns such as lipopolysaccharide (LPS) or inflammatory cytokines such as interferon *γ* (IFN*γ*), M*Φ* polarize to a proinflammatory, glycolytic macrophage phenotype M(LPS/IFN*γ*) activating distinct metabolic pathways [13, 14]. Cells produce huge amounts of ROS as toxic agents against host invading pathogens by the activation of the NADPH oxidase 2 complex (NOX2) [15, 16]. In contrast, for resolution of inflammation, wound repair and a return to homeostasis is needed. Therefore, anti-inflammatory shaped cells are recruited by the immune system releasing anti-inflammatory mediators like interleukin 4 (IL4) [17]. This cytokine polarizes M*Φ* into a M(IL4) phenotype to allow an optimal energy recovery via an aerobic metabolism [18]. As a result, these cells are disabled to generate high levels of ROS as defense mechanisms and use ROS only as signaling molecules in metabolic pathways [19]. Thus, the two distinct M*Φ* phenotypes M(LPS/IFN*γ*) and M(IL4) feature different redox requirements.

Therefore, we were interested to determine posttranslational modifications of PPAR*γ* based on the cellular redox milieu in the cytosol and the nucleus of LPS/IFN*γ* and IL4 polarized M*Φ*.

## 2. Material and Methods

### 2.1. Chemical Reagents

Cell culture media were purchased from Gibco (Carlsbad, USA), and their supplements provided by PAA Laboratories GmbH (Cölbe, Germany). Prokaryotic medium and supplements were ordered from Clontech (Takara, Japan) and Roth GmbH (Karlsruhe, Germany). Auranofin (AF), LPS, tetradecanoylphorbol acetate (TPA), hydrogen peroxide (H_2_O_2_), dimethylsulfoxide (DMSO), and the PPAR*γ* ligands rosiglitazone and 2-chloro-5-nitrobenzanilide (GW9662) were purchased from Sigma-Aldrich (St. Louis, USA). IFN*γ* was ordered from BioVision Inc. (Milpitas, USA) and IL4 from Peprotech (Hamburg, Germany). Enzymes were obtained from New England Biolabs (Ipswich, UK) and polymerases from Agilent Technologies Deutschland GmbH (Böblingen, Germany) and Clontech (Takara, Japan). Further chemicals, if not indicated otherwise, were ordered from AppliChem GmbH (Darmstadt, Germany), Merck KGaA (Darmstadt, Germany), Promega GmbH (Mannheim, Germany), Roche Diagnostics (Basel, Switzerland), and Sigma-Aldrich.

### 2.2. Cell Culture

The murine J774A.1 [20] M*Φ* cells were cultured in RPMI1640 and human HEK293T [21] cells in DMEM with high glucose at 37°C in a humidified atmosphere of 5% CO_2_. Both media were supplemented with 100 U/ml penicillin, 100 *μ*g/ml streptomycin, and 10% (*v*/*v*) heat-inactivated fetal calf serum. Cells were seeded 24 h before treatment in fresh medium.

M*Φ* were polarized by a combination of LPS (1 *μ*g/ml) and IFN*γ* (10 U/ml) into M(LPS/IFN*γ*) or by IL4 (10 ng/ml) into M(IL4) up to 24 h. Furthermore, cells were stimulated by TPA (1 *μ*M, 30 min), AF (3 *μ*M, 40 min), H_2_O_2_ (1 *μ*M–10 mM, 15 min), or DTT (100 *μ*M, 5 min) to induce redox stress. Treatments by PPAR*γ* ligands rosiglitazone (1 *μ*M) and GW9662 (10 *μ*M) were carried out for 24 h.

For N*-*ethylmaleimide (NEM) cysteine labeling of total cellular protein content, cultured cells were incubated in NEM (20 mM in RPMI medium) for 10 min on ice, washed 3 times in PBS before cell harvesting, and freezing in liquid nitrogen. Cell pellets were stored at -80°C until further processing.

### 2.3. Cloning of Expression Vectors

Expression vectors for stable overexpression of the proteins Clover-PPAR*γ*, roGFP2, and Grx1-roGFP2 were generated by replacing EGFP of pHR′SIN-cPPT-SE [22] by the appropriate coding sequences due to In-Fusion® (Clontech, Takara, Japan) recombination as previously described [23]. In brief, the plasmids pcDNA3-Clover [24], pDsRed-Monomer-C1-hPPAR*γ*1 [25], and pQE-60_Grx1-roGFP2 [26] were used as templates. N- and C-terminally HA-tagged hPPAR*γ* was generated by the same approach, elongated by an IRES sequence derived from the pLVX-TRE3G-mCherry (Clontech), and an additional Clover-sequence to generate a hPPAR*γ*-IRES-Clover expression construct. Therefore, h*PPARγ* was fused to the *HA*-coding sequence 5′-ATGTACCCATACGATGTTCCAGATTACGCT-3′ at the N-terminus and to 5′-CCCCCTCCGCCCCCACCTTACCCATACGATGTTCCAGATTACGCTTGA-3′ at the C-terminus. Cysteine to alanine mutants of hPPAR*γ* were created by mutagenesis of the TGX into GCX codons using PfuII polymerase (Agilent Technologies Deutschland GmbH, Böblingen, Germany). The same mutagenesis approach was used for the generation of serine to alanine (TCT to GCT) and serine to glutamic acid (TCT to GAG) mutants of hPPAR*γ*.

### 2.4. Transfection, Lentiviral Transduction, and Cell Sorting

To produce stably overexpressing cell lines, lentiviral particles were used which were generated by JetPRIME® (PEQLAB Biotechnologie GmbH, Erlangen, Germany) transfection of HEK293T with the lentiviral vectors psPAX2 (Addgene#12260), pMD2.G (Addgene#12259), and cloned vectors as previously described [23]. Transduced J774A.1 cells were sorted for green positive cells using BD FACSAria™ III cell sorter (BD Biosciences, Heidelberg, Germany).

### 2.5. Flow Cytometry Measurements

Flow cytometry measurements were carried out using a BD FACS LSRFortessa™ II flow cytometer (BD Bioscience, Heidelberg, Germany) coupled to the FACS Diva™ 6.1.3 software. Fluorescences of 1 x 10^4^ cells were detected and the means evaluated using FlowJo V10 software (FlowJo, Ashland, USA). The fluorescences of M*Φ* expressing *roGFP2*, its fusion constructs *Grx1-roGFP2*, and *roGFP2-PPARγ* were monitored at *λ*_ex_ of 405 nm and 488 nm, and the ration of 405/488 nm was calculated as relative fluorescence intensity (RFI). For the analysis, redox stimulated M*Φ* were washed with PBS, harvested, and analysed in PBS. To ensure the best possible comparability of cells treated by different concentrations of H_2_O_2_, M*Φ* were harvested before treatment, divided into subprobes, and inoculated with 1 *μ*M to 10 mM H_2_O_2_ in PBS on ice in parallel. To identify immediate redox modifications in response to possible redox stress, fluorescence of untreated cells, washed and resuspendend in PBS, was analysed before stimulation for 2–5 min. Then, the redox stimulus was added to the tube, thoroughly but gently mixed, and the fluorescence was determined until a constant value was accomplished.

### 2.6. PPAR*γ*-Dependent Transactivation Assay

PPAR*γ*-dependent transactivation assays were performed after cotransfection of *Clover-hPPARγ* encoding vectors together with the *Firefly* and *Renilla* luciferase genes containing vectors p(AOX)_3_-TK-Luc [27, 28] and pRL-CMV (Promega GmbH) as previously described [23]. In parallel to the Clover-hPPAR*γ* wild type construct, also cysteine to alanine mutants regarding hPPAR*γ* (C109A, C112A, C126A, C129A, C146A, C150A, C160A, C163A, C168A, and C284A) was used. Transactivation was measured using a 96-well plate format in a Mithras LB940 multimode reader (Berthold Technologies, Bad Wildbad, Germany). Relative luminescence units (RLU) were calculated in a dual luciferase approach, where Firefly fluorescence was normalized to Renilla. Transfection efficiencies of all vectors (PPAR*γ* wt and mutants) were normalized to Renilla fluorescence as well.

### 2.7. Subcellular Fractionation of Cytosolic and Nuclear Extracts

For total protein extracts, cells were lysed in a buffer (pH 7.4) containing 6.65 M urea, 10 mM Tris/HCl, 10% (*v*/*v*) glycerol, and 1% (*w*/*v*) sodium dodecyl sulfate (SDS), supplemented by 0.02 U/*μ*l Benzonase® nuclease (Merck KGaA, Darmstadt, Germany) for 1 min at room temperature.

To separate the cytosolic and nuclear protein fractions, cells were lysed by cytosolic extraction (CE) buffer (10 mM HEPES, 10 mM KCl, 0.1 mM EDTA, 0.3% (*v*/*v*) NP-40, pH 7.9) for 5 min at 4°C and multiple mixing times using 5x buffer volume of the cell pellet volume. After centrifugation (845 g, 5 min, 4°C), the supernatant was taken as cytosolic protein fraction. The sediment was washed 2 times with CE buffer without NP-40 and then treated with nuclear extraction buffer (20 mM HEPES, 400 mM NaCl, 1 mM EDTA, 25% (*v*/*v*) glycerol) for 10 min at 4°C using 1x buffer volume of sediment pellet volume. After centrifugation (18000 g, 5 min, 4°C), the supernatant was used as a nuclear protein fraction. All buffers contained 1x protease inhibitor (Roche Diagnostics, Basel, Switzerland) and 1 *μ*M phenylmethylsulfonyl fluoride (PMSF) to prevent protein degradation.

### 2.8. Immunoprecipitation

Frozen pellets of J774A.1 (3 x 10^7^ cells) overproducing N- and C-terminal HA-tagged hPPAR*γ* were lysed in lysis buffer (500 mM NaCl, 1% (*v*/*v*) NP-40, 50 mM Tris HCl pH 7.5, 5 mM EDTA, 0.5% (*w*/*v*) sodium deoxycholate, 0.1% (*w*/*v*) SDS), supplemented with 1x protease inhibitor (Roche Diagnostics) and PMSF (1 *μ*M), for 15 min at 4°C under rotated conditions. Lysates were additionally sonicated using a Branson Digital Sonifier® (Branson Ultrasonics, Eemnes, Netherlands) at 10% amplitude alternating a 0.9 sec pulse and 0.6 sec break for a total of 10 sec. After 10 min centrifugation (21000 g, 4°C), the supernatants containing the proteins were loaded on previously washed HA-beads (Roche Diagnostics) at a concentration of 4 mg/ml protein per 30 *μ*l bead slurry. The mixture was incubated overnight at 4°C on a rotator, and further precipitation was performed as described by the manufacturer.

For coimmunoprecipitation setups, cells were immediately processed after harvesting, lysed in 150 mM NaCl containing lysis buffer, and not sonicated.

### 2.9. Immunoblotting

The protein concentrations of total, cytosolic, and nuclear protein extracts were measured by the Lowry protein assay kit (Bio-Rad Laboratories GmbH, Munich, Germany). 100 *μ*g of total protein extracts was used for a standard 12% SDS polyacrylamide gel electrophoresis (SDS-PAGE) at 30 mA and 200 V, whereas only 50 *μ*g of the subcellular extracts was utilized. Therefore, lysates were supplemented with SDS sample buffer (50 mM Tris/HCl, 2% (*v*/*v*) glycerol, 2% (*v*/*v*) *β*-mercaptoethanol, 1.6% (*w*/*v*) SDS, 0.004% (*w*/*v*) Serva Blue G-250, pH 6.8) and boiled for 5 min at 95°C. After SDS-PAGE and 1.5 h blotting of the proteins onto an Amersham™ Protran™ nitrocellulose membrane at 1.8 mA/cm^2^ and 25 V, membranes were incubated with 5% (*w*/*v*) milk dissolved in TBS (50 mM Tris/HCl, 140 mM NaCl, pH 7.2) for 1 h at room temperature. For specific, immunologic labeling, primary antibodies against GFP (1 : 1000, ab1218, Abcam plc Cambridge, UK), HA (1 : 1000, 2367, Cell Signaling Technology, Danvers, USA), and PPAR*γ* D69 (1 : 1000, 2430, Cell Signaling Technology, Danvers, USA) were used in 5% (*w*/*v*) milk/TBS for 3 h at room temperature or 16–48 h at 4°C. Additionally *β*-actin anti-mouse (1 : 10000, A2228, Sigma-Aldrich), *β*-actin anti-rabbit (1 : 5000, A5441, Sigma-Aldrich), *β*-tubulin (1 : 5000, T-4026, Sigma-Aldrich), and lamin A/C E-1 (1 : 1000, Sc-376248, Santa Cruz, Dallas, USA) were used as protein loading controls. After 3 times washing in TBS-T (TBS supplemented with 0.05% (*v*/*v*) Tween-20), membranes were incubated for 1.5 h with IRDye*®* 680RD or 800CW donkey anti-mouse or donkey anti-rabbit (1 : 10000, 925-68072, 925-32212, 925-68073, 925-32213, LI-COR GmbH, Bad Homburg, Germany) as secondary antibodies, respectively. Immunological detections were carried out using an Odyssey® infrared imaging system (LI-COR GmbH). Blots were analyzed by Image Studio Digits Version 5.0 (LI-COR GmbH).

### 2.10. LC/MS Analysis

Immunoprecipitated HA-tagged hPPAR*γ* passed a SDS-PAGE, followed by a Coomassie staining of the SDS gel and the cutting out of gel piece with a size of approximately 58 kDa ± 3 kDa. The gel pieces were destained in 60% (*v*/*v*) methanol, 50 mM ammoniumbicarbonate (ABC), and washed in 50 mM ABC [29]. Proteins were reduced in 1 mM DTT, 50 mM ABC for 1 h at 56°C and alkylated for 45 min in 5 mM D5-NEM. Samples were digested for 16 h with LysC (sequencing grade, Promega GmbH) at 37°C in 50 mM ABC, 0.01% Protease Max (Promega GmbH), and 1 mM CaCl_2_. Peptides were eluted in 30% (*v*/*v*) acetonitrile and 3% (*v*/*v*) formic acid, centrifuged into a fresh 96 well plate, dried in speed vac, and resolved in 1% (*v*/*v*) acetonitrile and 0.5% (*v*/*v*) formic acid.

Liquid chromatography/mass spectrometry was performed on Thermo Scientific™ Q Exactive Plus equipped with an ultra-high performance liquid chromatography unit (Thermo Scientific Dionex Ultimate 3000) and a Nanospray Flex Ion-Source (Thermo Fisher Scientific GmbH, Dreieich, Germany). Peptides were loaded on a C18 reversed-phase precolumn (Thermo Fisher Scientific GmbH) followed by separation on a with 2.4 *μ*m Reprosil C18 resin (Dr. Maisch HPLC GmbH, Ammerbuch, Germany) in-house packed picotip emitter tip (diameter 100 *μ*m, 15 cm from New Objectives) using a gradient from 4% (*v*/*v*) acetonitrile, 0.1% (*v*/*v*) formic acid to 40% (*v*/*v*) eluent B (99% (*v*/*v*) acetonitrile, 0.1% (*v*/*v*) formic acid) for 30 min and a second gradient to 60% (*v*/*v*) B for 5 min with a flow rate 400 nl/min. MS data were recorded by data-dependent acquisition. The full MS scan range was 300 to 2000 m/z with resolution of 70000, and an automatic gain control value of 3 x 10^6^ total ion counts with a maximal ion injection time of 160 ms. Only higher charged ions (2+) were selected for MS/MS scans with a resolution of 17500, an isolation window of 2 m/z, and an automatic gain control value set to E5 ions with a maximal ion injection time of 150 ms. MS1 data were acquired in profile mode.

MS data were analysed by Peaks7 (Bioinformatics Solutions Inc.). Proteins were identified using the mouse reference proteome database UniProtKB with 52538 entries, released in 2/2018, supplemented with human hPPAR*γ* with a false discovery rate of 1%. The enzyme specificity was set to LysC with one unspecific end. NEM (+125.05), respectively, D5-NEM (+130.08) on cysteines, phosphorylation (+79.97) on serine, threonine and tyrosine, deamidation (+0.98) on asparagine, and glutamine and methionine oxidation (+15.99) were variable modifications. A second database search was applied to identify disulfides unpaired fragmentation (-2.02) together with methionine oxidation (+15.99) and deamidation (+0.98) on asparagine and glutamine as frequent modification.

### 2.11. Statistics

Each experiment was performed at least 3 times. All generated data of the same characteristics are shown as the mean ± standard deviation (SD). Statistics were carried out using either one- or two-way analysis of variance modified with Bonferroni multiple comparison test, respectively, unpaired and paired Student's *t*-test. The choice of analysis was adapted to the trial requirements. Significant differences were set as follows: ^∗^≙*p* ≤ 0.05; ^∗∗^≙*p* ≤ 0.01; ^∗∗∗^≙*p* ≤ 0.001.

## 3. Results

### 3.1. Cellular Redox Milieus of M(LPS/IFN*γ*)- and M(IL4)-Polarized M*Φ* Differently Induce Redox-Based PTMs of roGFP2

**Figure 1:**
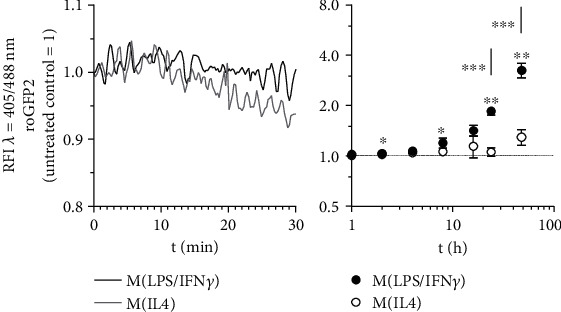
Redox status of macrophages during M(LPS/IFN*γ*) vs. M(IL4) polarization. J774A.1 cells were transduced by vectors encoding the redox-sensitive marker proteins roGFP2. To mimic macrophage polarization, cells expressing *roGFP2* were treated with 1 *μ*g/ml LPS combined with 10 U/ml IFN*γ* (M(LPS/IFN*γ*)) or with 10 ng/ml IL4 (M(IL4)). The redox status was determined by FACS analysis at 405 nm and 488 nm during the first 30 min ongoing (left panel, representative experiment) or at indicated hours (right panel, quantitative data) after polarization starts. All experiments were performed at least three times. Mean values ± SD are provided. Untreated control cells were set as 1. (^∗^*p* ≤ 0.05, ^∗∗^*p* ≤ 0.01, ^∗∗∗^*p* ≤ 0.001).

Using cellular overexpression of a redox-sensitive green fluorescent protein 2 (roGFP2), we monitored changes in the cellular redox milieu of differently polarized J774A.1 M*Φ*. Redox-based PTMs of roGFP2 changed the spectral properties of the fluorophore, which could be calculated by relative fluorescence intensities (RFI) at the ratio of 405/488 nm (Figure 1). A decrease of the RFI ratio after cell treatment compared to unstimulated control cells was interpreted to indicate reducing conditions, as observed following DTT treatment (Supplementary Figure 1). Oxidizing conditions are indicated by an increase of 405/488 nm, as detected in response to H_2_O_2_ stimulation. During the first 30 min of M(LPS/IFN*γ*) and M(IL4) polarization, we could not detect significant differences in the redox environment of roGFP2 cells (Figure 1, left panel). After these initial phases, M(LPS/IFN*γ*) polarization was characterized by a significant, exponential increase of oxidizing conditions in the cells indicated by a RFI of 1.02 (±0.02) after 1 h, 1.04 (±0.01) at 2 h, and 3.26 (±0.3) at 48 h. Although RFI ratios of M(IL4) macrophages reached also 1.01 (±0.02) after 1 h, they in contrast stayed at this level up to 24 h (1.06 ± 0.06). Only after 48 h, a small, but not significant increase to 1.30 (±0.14) could be detected. In parallel, we checked the mRNA expression of polarization marker genes such as *iNOS* for M(LPS/IFN*γ*) and *Arg1* for M(IL4) (Supplementary Figures 2A and 2B). Altogether, these data indicate that redox-sensitive GFP2 is suitable to recognize and indicate redox changes in M*Φ*, allowing to discriminate two M*Φ* phenotypes M(LPS/IFN*γ*) and M(IL4). In this regard, classical activation by LPS/IFN*γ* of M*Φ* causes a more oxidizing redox milieu. On the contrary, IL4 activation points towards reducing conditions.

### 3.2. Polarization of M*Φ* Affects the Redox Status of Nuclear Localized roGFP2-PPAR*γ*

**Figure 2:**
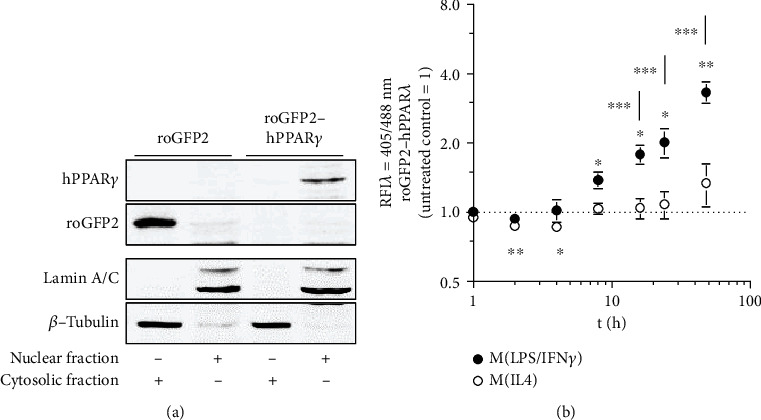
Redox status of polarizing M*Φ* affects proteins located in the nucleus. After cloning of a hybrid protein of the redox marker roGFP2 and the nuclear receptor hPPAR*γ*, this construct was transduced into J774A.1 M*Φ* (a). J774A.1 M*Φ* transduced with roGFP2 were used as control. Cellular fractionation (a) was performed to analyze cellular localization of roGFP2 and roGFP2-hPPAR*γ*. J774A.1 M*Φ* expressing roGFP2-hPPAR*γ* was treated for the indicated times with LPS/IFN*γ* or IL-4, and the redox status was determined by FACS analysis at 405 nm and 488 nm. Quantification of flow cytometric data is provided in (b). All experiments were performed at least three times. Mean values ± SD are provided. Untreated control cells were set as 1. (^∗^*p* ≤ 0.05, ^∗∗^*p* ≤ 0.01, ^∗∗∗^*p* ≤ 0.001).

Based on the roGFP2 data, we were interested, whether these different redox conditions during M*Φ* polarization might affect PPAR*γ*. Taking into consideration that PPAR*γ* is a nuclear receptor, known to alter the expression of target genes by binding to their promoter regions, we first analyzed whether ROS in M*Φ* are ubiquitously present or restricted to the cytosol. Therefore, J774A.1 M*Φ* were transduced with a vector encoding for roGFP2 coupled to hPPAR*γ*. We assumed that this setup enables the resulting hybrid protein to localize similarly to endogenous mPPAR*γ*. After verifying the functionality of expression plasmids (data not shown), cellular localization of roGFP2-hPPAR*γ* was determined by cytosolic and nuclear fractionation. In line with our presumptions, cells transduced with this *roGFP2-hPPARγ* construct (Figure 2(a), last lane), showed nuclear localization of the fusion protein, only. In contrast, roGFP2 was located in the cytosol (Figure 2(a)). Using J774A.1 M*Φ* expressing roGFP2-hPPAR*γ*, we next determined whether M(LPS/IFN*γ*) or M(IL4) might alter the redox status of proteins located in the nucleus. Interestingly, a maximum of reducing conditions was confirmed after 2 h of treatment with either of the two polarization settings. Specifically, M(LPS/IFN*γ*) stimulation reduced values to 0.94 (±0.04) and M(IL4) to 0.87 (±0.02). LPS/IFN*γ* significantly increased the relative fluorescence intensity of roGFP2-hPPAR*γ* cells starting at 4 h (1.02 ± 0.12) staying above control cells and IL4 polarized cells (Figure 2(b)). After 48 h, the RFI ratio increased to 3.33 (±0.36). In parallel, in IL4-treated roGFP2-hPPAR*γ* cells the RFI ratio was 0.87 (±0.03) after 4 h and slightly increased to 1.34 (±0.28) after 48 h. Initiation of polarization was validated by analyzing mRNA expression of marker genes, i.e., *iNOS* for M(LPS/IFN*γ*) and *Arg1* for M(IL4) (Supplementary Figures 2C and 2D).

### 3.3. Cysteines of PPAR*γ* Are Targets of Redox-Based PTMs

Having successfully established that redox stress-induced PTMs in cytosolic and nuclear located roGFP2 (Supplementary Figure 3), we determined whether cysteines of hPPAR*γ* are also modified. As a first step, we treated HA-tagged hPPAR*γ* overexpressing J774A.1 cells under polarizing conditions, i.e., redox stress-inducing agents, and performed LC/MS analysis of immunoprecipitated hPPAR*γ*. H_2_O_2_ was used as a direct ROS source. Auranofin is an inhibitor of the thioredoxin reductase, thus blocking the cellular antioxidative protection system, which leads to an increased oxidizing cell milieu (Supplementary Figure 3). TPA finally is an indirect inducer of cellular ROS via activating the NOX2 complex. For the detection of redox-modified cysteines, cellular existing reduced thiols were labeled with NEM before cell harvesting to ensure the fixation of the protein redox status under cellular conditions. On the contrary, oxidized cysteines were labeled with D5-NEM during sample reprocessing. LC/MS identified specific peptides covering 65% of immunoprecipitated hPPAR*γ* protein (Supplementary Figure 4) including nine out of the ten cysteines (Table 1), which could be detected with the labeling agents NEM/D5-NEM as well as disulfides in MS2 spectra (Supplementary Figures 5–9).

**Figure 3:**
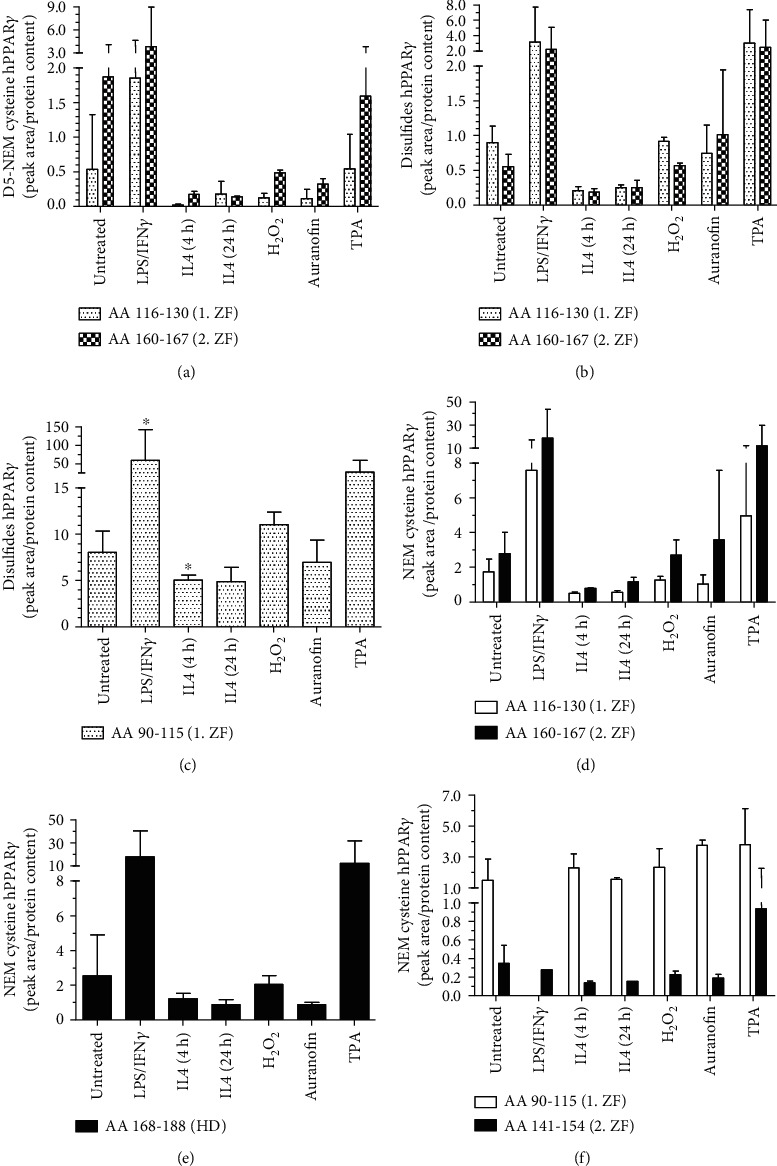
Determination of the redox status of hPPAR*γ* cysteines via mass spectrometric analysis. J774A.1 cells were transduced using lentiviral particles with integrated N- and C-terminal HA-labeled hPPAR*γ*. To induce redox stress, M*Φ* were treated by 1 *μ*g/ml LPS combined with 10 U/ml IFN*γ* for 4 h, 10 ng/ml IL4 for 4 h and 24 h, H_2_O_2_ (100 *μ*M, 15 min), auranofin (3 *μ*M, 40 min), or TPA (1 *μ*M, 30 min). Untreated cells were used as controls. The redox states of reduced cysteines were fixed by NEM before cell harvest on ice. HA-tagged hPPAR*γ* was HA-immunoprecipitated, existing oxidized cysteines denatured and labeled by D5-NEM. Peptides were analysed by LC/MS after LysC digestion. Quantification of cellular oxidized (D5 NEM, disulfides) and reduced (NEM) cysteine each with respect to the corresponding protein concentrations of the sample (Supplementary Figure 4) are mapped (a–f). Peptides of different hPPAR*γ* amino acid sequences (AA) are depicted in Table 1. Cysteines affiliations to the first and second zinc finger (ZF) and hinge domain (HD) are indicated. All experiments were performed at least three times. Mean values ± SD are provided. (^∗^*p* ≤ 0.05).

Interestingly, we detected enhanced oxidization of cysteines (D5-NEM) only within the amino acid fragments AA 116-130 and AA 160-167, as members of the first and second zinc finger regions of hPPAR*γ* (Figure 3(a)). More strikingly, only LPS/IFN*γ* and TPA led to the redox-based PTMs, whereas the typical redox stress inducers H_2_O_2_ and auranofin did not. The inspection of available disulfides of these amino acid fragments together with those of the first half of the first zinc finger motif pointed to a similar direction, allowing inference of an uncompleted labeling of oxidized cysteines by D5-NEM (Figures 3(b) and 3(c)). Surprisingly, reduced cysteines (NEM) of the peptides AA 116-130 and AA 160-167 following LPS/IFN*γ* and TPA stimulations were also increased (Figure 3(d)). The quantification of the heavy (D5-NEM) to light (NEM) ratios confirmed a linear correlation of both labelings (Supplementary Figure 10). This suggested a treatment dependent accessibility of the cysteines in the second part of both zinc fingers due to the NEM labeling before the cell harvest, which would be the first step of a subsequent oxidization. The data of AA 168-188 support these assumptions showing a similar PTM response of a reduced cysteine 168 inside the hinge domain of hPPAR*γ* (Figure 3(e)). Complementing our data, IL4 treatment for 4 h and 24 h resulted in the lowest detection rate of oxidized and reduced cysteines referring to a previously established reducing redox milieu in the stimulated cells (Figures 3(a)–3(e)). However, cysteines within the first part of both zinc finger domains of hPPAR*γ* (AA 90-150, AA 141-154) showed almost no differences in the detection level of NEM-labeled cysteines in response to any induced redox stress (Figure 3(f)). Nevertheless, redox-based posttranslational modifications within parts of the DNA-binding domain of hPPAR*γ* were detected. Finally, the absence of H_2_O_2_ induced PTMs at cysteines of hPPAR*γ* despite induced redox stress in the cells was validated via BIAM switch assay using the cysteine modifications of peroxiredoxin 3 (Prx3) as control for the chosen treatments (Supplementary Figure 11).

### 3.4. Mimicking Redox-Based PTMs of hPPAR*γ* Cysteines Point to an Altered DNA-Binding Capacity

**Figure 4:**
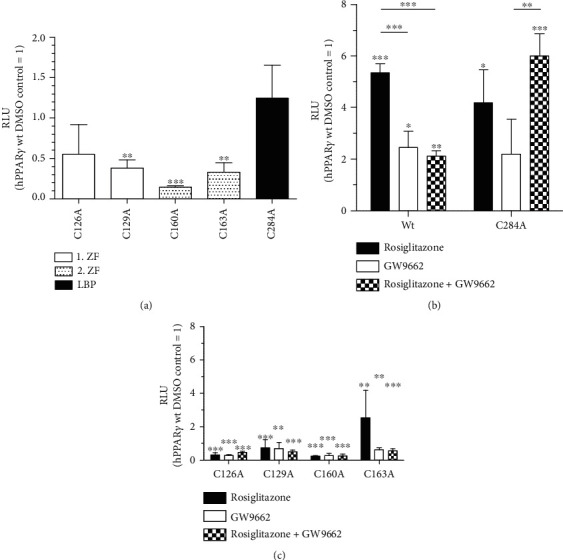
Site-directed amino acid exchange of hPPAR*γ* cysteines inhibits PPAR*γ*-dependent transactivation. A PPAR*γ*-dependent transactivator assay was used to verify the effect of LC/MS detected (C126, C129, C160, and C163) and potential (C284) redox-modified hPPAR*γ* cysteines. Therefore, HEK293T cells were transiently transfected with vectors encoding hPPAR*γ* wt and cysteine-to-alanine mutants and a *Firefly* luciferase under the control of a PPAR*γ*-responsive element as promoter. Affiliation of the cysteines towards zinc finger domain (ZF) and ligand-binding pocket (LBP) are depicted. Cells were cotransfected with a *Renilla* luciferase vector with a permanent active promoter cloned in front of the gene, so that relative luminescence units (RLU) were calculated in a dual luciferase assay. Transfected cells were stimulated for 24 h with DMSO (a), rosiglitazone (1 *μ*M), and GW9662 (10 *μ*M) alone or in combination (b, c). Mean values of three individual experiments ± SD are provided. DMSO treated hPPAR*γ* wt cells were set as 1. (^∗^*p* ≤ 0.05, ^∗∗^*p* ≤ 0.01, ^∗∗∗^*p* ≤ 0.001).

To validate a putative role of identified redox-mediated PTMs of hPPAR*γ* cysteines located in the zinc finger regions of the protein's DNA-binding domain (DBD), we asked whether these modifications were associated with functional changes in hPPAR*γ*-dependent transactivation. We performed a transient PPAR*γ* transactivator assay based on a dual luciferase system. To analyse the role of modified cysteine residues located in the DBD, we mutated cysteines to alanines of all LC/MS verified oxidized cysteines of hPPAR*γ* (C126A, C129A, C160A, and C163A) (Figure 4). In addition, a mutant of the nonredox determined cysteine 284 was generated (C284A). Interestingly, the basal level of luciferase activity of all four cysteine-containing mutations within the zinc finger regions was decreased in comparison to wildtype (wt) hPPAR*γ* (Figure 4(a)). In contrast, relative luminescence units (RLU) by the mutant C284A were slightly, but not significantly, increased. To further examine differences between the mutants and the wildtype constructs, cells were treated for 24 h with PPAR*γ* ligands. Rosiglitazone represents a PPAR*γ* agonist, which strongly increased the transactivator activity of wt hPPAR*γ* (Figure 4(b)). In contrast, the antagonist GW9662 only slightly increased transactivation of wt hPPAR*γ*, but at the same time blocked agonist, i.e., rosiglitazone-dependent transactivation. GW9662 treatment of cells expressing the C284A mutant led to a similar transactivation as observed for wt hPPAR*γ*, but surprisingly, stimulation with the agonist rosiglitazone was not blocked. Therefore, a putative redox-modification of cysteine C284 located in the ligand-binding pocket of hPPAR*γ*, which could not be verified via MS due to the lack of peptide coverage, could be envisioned to alter ligand binding of the transcription factor. In line with our expectation, the cysteine to alanine mutants located in the DBD of hPPAR*γ* completely blocked hPPAR*γ* transactivation (Figure 4(c)). Only hPPAR*γ* C163A expressing cells responded with enhanced luciferase activity to rosiglitazone stimulation. These data support a connection between cysteine oxidation within the zinc finger region of hPPAR*γ*, provoking a loss of the DNA-binding capacity, consequently altering the transactivating function of the transcription factor.

### 3.5. Phosphorylation of Serine 82 of hPPAR*γ* Is a Prerequisite for Redox-Based PTMs Initiation

**Figure 5:**
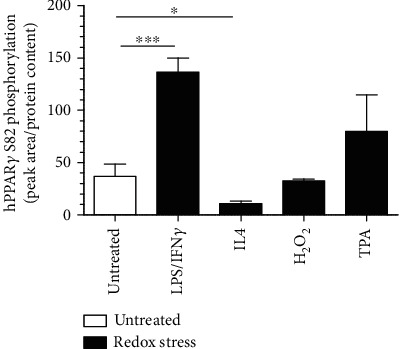
hPPAR*γ* phosphorylation states and its connection to redox-modification. A quantification (mean values ± SD) of hPPAR*γ* serine S82 phosphorylation of reanalysed LC/MS data sets of HA-immunoprecipitated HA-tagged hPPAR*γ* expressed in J774A.1 cells following different treatments (1 *μ*g/ml LPS combined with 10 U/ml IFN*γ* for 4 h, 10 ng/ml IL4 for 4 h, 100 *μ*M H_2_O_2_ for 15 min, 1 *μ*M TPA for 30 min) as indicated is provided. Mean values of three individual experiments ± SD are provided. (^∗^*p* ≤ 0.05, ^∗∗∗^*p* ≤ 0.001).

Considering missing oxidation of cysteines of hPPAR*γ* following H_2_O_2_-treatment, we checked whether nonredox-based PTMs like phosphorylations might correlate with our previous findings. Therefore, we reanalysed the LC/MS data collection of redox-modified hPPAR*γ* for phosphorylations at serine-, tyrosine-, and threonine-residues. As shown in Figure 5, LPS/IFN*γ* stimulation significantly increased the phosphorylation of serine 82 (S82) of hPPAR*γ*, which was also shown in TPA-treated cells. On the contrary, following IL4 stimulation minimal phosphorylation of S82 was detected. H_2_O_2_ was without effect. Following these lines, phosphorylation of S82 might be linked to the initiation of redox-based PTMs at the cysteines of hPPAR*γ* because both MS data setups showed the same dependence on the stimulus.

### 3.6. Redox-Modifications of hPPAR*γ* Altered Binding to VIM

**Figure 6:**
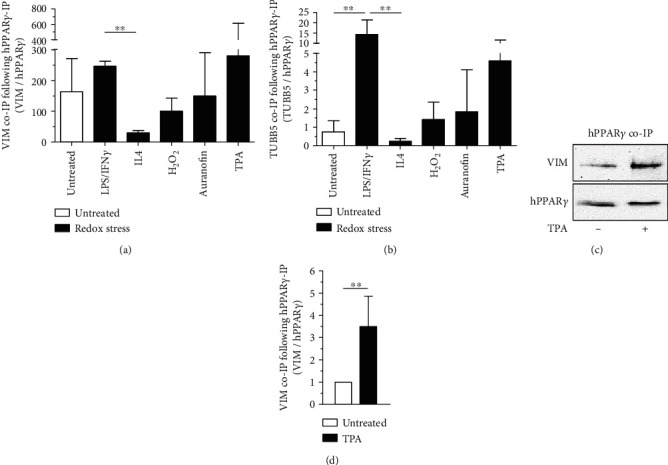
Protein-protein interaction of redox-modified hPPAR*γ*. The LC/MS data sets of HA-immunoprecipitated HA-tagged hPPAR*γ* with and without redox stress treated J774A.1 cells (1 *μ*g/ml LPS combined with 10 U/ml IFN*γ* for 4 h, 10 ng/ml IL4 for 4 h, 100 *μ*M H_2_O_2_ for 15 min, 1 *μ*M TPA for 30 min) were reanalysed for hPPAR*γ* coimmunoprecipitated proteins. Quantification of identified VIM (a) and TUBB5 (b) content via LC/MS is depicted. To verify LC/MS data, J774A.1 cells producing HA-tagged hPPAR*γ* were treated with TPA (1 *μ*M, 30 min) and coimmunoprecipitation for HA-hPPAR*γ* was performed. Lysates of untreated cells were processed in parallel. All experiments were performed at least three times. (c) shows a representative Western blot analysis and quantification of mean values ± SD is provided in (d). (^∗∗^*p* ≤ 0.01).

Considering that PPAR*γ* is known as a regulator with dual functionality as a DNA-binding transcription factor as well as a protein interaction partner in a DNA-unbound state, we focused our interest on PPAR*γ* interaction partners, following classical LPS/IFN*γ* vs. alternative IL4 M*Φ* activation. Therefore, we reanalysed the LC/MS data of HA-immunoprecipitated hPPAR*γ* to search for coimmunoprecipitated proteins. We identified vimentin (VIM) and tubulin *β*-5 chain (TUBB5) as known proteins of the cellular cytoskeleton (Figure 6). The highest levels of coimmunoprecipitated VIM (Figure 6(a)) and TUBB5 (Figure 6(b)) proteins were observed after LPS/IFN*γ*- and TPA-treatment. Lowest binding was found in response to IL4-stimulation. An enhanced hPPAR*γ*-protein interaction after TPA stimulation could be verified for hPPAR*γ*-VIM interaction by Western blot analysis (Figures 6(c) and 6(d)). These data finally suggested an enhanced protein-protein interaction of modified hPPAR*γ* with proteins of the cytoskeleton.

## 4. Discussion

M*Φ* are essential sentinels of the immune system, which are involved in direct host defenses against invading pathogens, but also in regulating various immunological processes [30]. Therefore, M*Φ* can polarize into different phenotypes by drastically changing their metabolism and cellular redox status to establish optimal cellular conditions to fulfill their purposes in the host [13, 14].

Therefore, we first characterized the cellular redox milieu of M(LPS/IFN*γ*) and M(IL4) polarized M*Φ* regarding their capability to induce redox-based PTMs at the cysteines of cellular expressed roGFP2 as a redox marker protein. We observed that M*Φ* phenotypes established oxidizing or reducing conditions after a short initial phase depending on the used polarization regime (Figure 1, Supplementary Figure 2). After recognition of pathogen-associated molecular patterns like LPS/IFN*γ*, macrophages start to polarize more and more into the host defensive phenotype by a complex shift from an aerobe to a more anaerobic metabolism. This is accomplished by increased energy generation via glycolysis, the pentose phosphate pathway, and a shortened citrate cycle with an inhibited respiratory chain [14]. At the same time, the cellular redox status is increased by using generated nicotinamide adenine dinucleotide phosphate (NADPH) as a cofactor for the generation of reactive oxygen species (ROS) in the form of ∙O_2_^−^ by the NADPH oxidase 2 (NOX2) [31]. This radical can be converted by the cells into reactive substances such as hydrogen peroxide (H_2_O_2_), hypochlorous acid (HOCl), and N-chloramine (RNCl), which together with NO are used to kill microorganisms [15]. Following those lines, the potential oxidizing cell milieu of classically LPS/IFN*γ* activated M*Φ* appeared to be significantly enhanced in our experiments compared to alternatively IL4-activated cells, which are not qualified to produce high levels of ROS due to their lack of NOX2 activation and expression [18, 19, 32]. Nevertheless, the development of specific oxidation levels of the cellular redox milieu is a time-consuming process and highly dependent on the polarization status of the macrophages.

Low concentrations of ROS are omnipresent in cells as signaling molecules and can freely cross cell membranes. Therefore, nuclei possess a separated antioxidative protection system, which shields the DNA against the harmful oxidative effects of ROS [33]. As a result, thioredoxin 1 (Trx1) is imported into the nucleus in response to oxidative stress and in parallel oxidized redox buffers from inside the nuclear compartment are continuously exported into the cytosol [34, 35]. This leads to reduced redox potentials and conveys a relative resistance towards oxidations in the nuclei. In order to verify this relationship, we used hydrogen peroxide (H_2_O_2_) as a direct oxidant, as well as auranofin and TPA in our studies. The use of auranofin and TPA should only indirectly lead to changes in the redox balance of the cells, as both substances modulate metabolic pathways and activity of redox-relevant proteins. Thus, auranofin acts as a specific inhibitor of selenoproteins such as thioredoxin reductase (TrxR) and thioredoxin-glutathione reductase (TGR), weakening antioxidative protective mechanisms of cells [36]. TPA, on the other hand, leads to a translocation of protein kinase C to the cell membrane, allowing phosphorylation of NOX2 subunits. As a result, the functional NOX2 complex can form, which is capable of producing ROS [37]. H_2_O_2_, auranofin, and TPA as short term redox stress inducers attenuated the oxidation effect in cells expressing a nuclear located fluorophore pointing towards an active antioxidative defence in the nucleus independent from the protection in the cytosol (Supplementary Figure 3). Classically, LPS/IFN*γ* activation of M*Φ* increased the oxidative environment in the nucleus at later time points, which was even stronger than that of the cytosolic redox status (roGFP2-hPPAR*γ* vs. roGFP2, Figure 2(d) vs. Figure 1(a)). This implicates a specific inactivation of the nuclear protection system, which is reasonable considering the desired oxidation of many nuclear proteins. In contrast, M(IL4) polarization leads to an extension of reducing conditions during the first hours in the nucleus. During polarization, M*Φ* try to adapt as fast as they can to tissue-specific requirements and needs. Therefore, an amplification of a specific redox signal, independently whether oxidative or reductive, could be beneficial to alter the function of many different transcriptional regulations at the same time by redox-based PTMs. Following those lines, we verified differences in the cytosolic and nuclear redox environment of redox challenged M*Φ*, suggesting that redox-based PTMs of hPPAR*γ* can occur.

PPAR*γ* is a transcription factor involved in many cellular processes related to fatty acid metabolism and also M*Φ* polarization [38–41]. Its broad spectrum of capabilities is maintained by the dual functionality of the protein in altering transcription of genes DNA-dependently and –independently [7, 8, 42]. Its activity is controlled by alterations of its conformation, which are initiated by PTMs of amino acids throughout the protein and in response to specific ligand binding to its ligand-binding domain (LBD) [43, 44]. As a consequence, the interaction profile of hPPAR*γ* is shifted, characterized by supporting or suppressing distinct protein-protein interactions. Thus, phosphorylation, SUMOylation, acetylation, ubiquitylation, and O-GlcNAcylation were already identified without a connection to possible redox-based modifications [44]. Therefore, we focused our analyses towards amino acid alterations of this transcription factor, triggered by changes of the redox environment in M(LPS/IFN*γ*) and M(IL4)-polarized macrophages. To connect observations to chemical characteristics of a changing cellular redox milieu, the effects of redox stress-inducing agents like H_2_O_2_, auranofin, and TPA were monitored in parallel via mass spectrometry. Interestingly, H_2_O_2_ and auranofin did not oxidize the cysteines of the transcription factor, whereas oxidation of cysteines (disulfides and D5-NEM, Figure 3) in the zinc finger motifs of hPPAR*γ*s DNA-binding domain was increased after TPA or macrophage polarization into the M(LPS/IFN*γ*) phenotype. In line, M(IL4)-polarized macrophages showed less oxidized cysteines. These data indicate that hPPAR*γ* is oxidized by the cellular redox environment, but its initiation is most likely coupled to a redox-independent process. As shown by our roGFP2 experiments, TPA induces only a weak cellular oxidizing condition compared to the other used oxidants. However, its mediated ROS generation is not only accompanied by the formation of an active NOX2 complex, but also by phosphorylation and activation of protein kinase C (PKC) and mitogen-activated protein kinase (MAPK) pathways [45, 46].

The latter is known to be also involved in the well-characterized phosphorylation of hPPAR*γ* at serine S82 (S112 for hPPAR*γ* isoform 2) [47–50]. Analyzing the phosphorylation status of the transcription factor in our system revealed a correlation between phosphorylation of S82 (pS82) and cysteine oxidations (Figure 3 vs. Figure 5). In general, a phosphorylation in close proximity to a cysteine promotes the thiol state of an amino acid residue and therefore decreases its oxidative sensitivity by electrostatic modulation [51]. But S82 of hPPAR*γ* is located within the ligand-independent activator domain AF1 of the protein and thus separated from the cysteine-containing DBD. Furthermore, phosphorylation of S82 has been shown to cause extensive conformational changes of hPPAR*γ* altering the transactivator activity of the protein [52]. This is executed by an intramolecular interaction of the AF1 domain to the LBD. This supports our hypothesis of a coupled mechanism of phosphorylation, shifting the tertiary structure of the protein and probably also the accessibility of amino acids, introducing cysteine oxidation. To identify oxidized cysteines methodically by MS, we fixed the redox status of all reduced cysteines by NEM-labeling before the cells were harvested. Interestingly, we detected different levels of NEM cysteines, which were only elevated during TPA- and LPS/IFN*γ*-stimulation of M*Φ*, simultaneously to the already mentioned cysteine oxidations and S82 phosphorylation. This mechanism points towards a differential accessibility of hPPAR*γ* cysteines under cell culture conditions, which are supported by the changes of the protein structure due to a phosphorylation as described earlier. Especially the data of C168 inside the AA 168-188 peptide of hPPAR*γ* with its amino acid located inside the flexible hinge domain of the transcription factor fosters our assumption of a stimulation-dependent differential accessibility of cysteines in the protein. In this context, the identification of reduced in parallel to oxidized cysteines appears plausible. Reduced thiols inside the DBD are not bound to zinc and therefore the protein is already in a conformation facilitating further oxidation.

**Figure 7:**
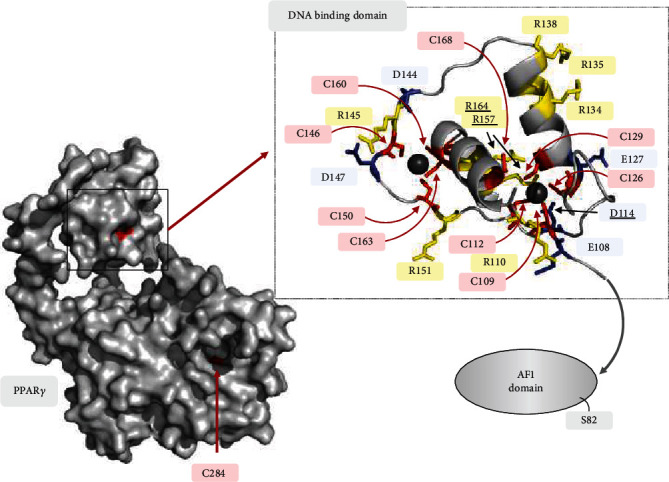
Amino acid environment of hPPAR*γ* cysteines. Depicted hPPAR*γ* crystal structure is based on PDB ID: 3DZY [53]. Cysteines are highlighted in red, arginines in yellow, and glutamic acids as well as aspartic acids in blue. Amino acids that could directly influence the redox status of cysteines are underlined. Zinc complexed by the cysteines is characterized by black spheres. The location of the AF1 domain missing in the structure with S82 contained therein is indicated by a grey arrow. The localization of C284, whose redox status could not be detected in the LC/MS, is indicated in the overall structure of hPPAR*γ*.

Other factors that could also control the redox state of hPPAR*γ* cysteines are the local electrostatic effects of amino acids in the immediate vicinity of the residue [51]. Therefore, negatively charged side chains of glutamic and aspartic acid decrease oxidative sensitivity of cysteines by promoting the neutral thiol state. In the contrary, the negatively charged thiolate state of cysteines can be provoked and stabilized by the positively charged side group of arginine, increasing the oxidative sensitivity of the cysteine residue. A closer look reveals that there are many charged amino acids in close proximity to cysteines inside the DBD of hPPAR*γ* (Figure 7). However, for most of these amino acids, an influence on the cysteines' redox sensitivity can be excluded due to the position of the side chains. Interestingly, the side chains of R157 and R164 protrude directly into the reactive centres of the Zn^2+^-complexing cysteines. From this, it can be assumed that cysteine residue oxidation is favoured. On the other hand, D114 could also be involved in fine-tuning this redox regulation.

It has already been shown that zinc itself modulates different aspects regarding hPPAR*γ* for PPAR*γ* signaling [54, 55]. Therefore, the accessibility of cysteines could be additionally regulated via the zinc balance of the cells. Zinc homeostasis is discussed very controversially in the literature concerning macrophage polarization. Generally, zinc is known for its anti-inflammatory and antioxidative functions. In doing so, it attenuates the oxidative burst of macrophages following gram-positive *Staphylococcus aureus* infection and interferes with ROS production by inhibiting NADPH [56–58]. At the same time, zinc is essential for many proteins like PKC and MAPK, involved in different signaling and inflammation pathways of classical LPS/IFN*γ*-polarized macrophages [59, 60]. Thus, the zinc finger structures and the corresponding functions of hPPAR*γ* could also be influenced by the cellular availability of zinc. Further experiments have to clarify a cause -effect relationship.

Our data obtained from immunoprecipitated hPPAR*γ* derived from M(IL4)-polarized M*Φ* further support a coupled mechanism between phosphorylation, a conformational change, and oxidation of cysteines of the transcription factor. IL4 is known to stimulate PPAR*γ* activity in M*Φ* in a signal transducer and activator of transcription 6- (STAT6-) dependent manner [61, 62]. As a consequence, activated PPAR*γ* binds directly to DNA, and transcription of PPAR*γ* target genes is initiated without phosphorylation of S82 [48]. In line, our MS data showed lowest pS82 as well as minor oxidized cysteines in parallel to highest induction of the PPAR*γ* target gene Arg1 following IL4 stimulation (Figure 3 vs. Figure 5 vs. Supplementary Figure 2). Focusing on TPA treatment, transcriptional activity of PPAR*γ* is reduced, although phosphorylation of S82 per se did not reduce the DNA-binding activity of the protein [48]. Thus, the question is raised, whether oxidized cysteines of redox-modified hPPAR*γ* mediate transactivation changes. Our data, using PPAR*γ*-dependent transactivator assays analysing the transactivation of hPPAR*γ* wt and cysteine-to-alanine mutants, provided evidence that the loss of only one cysteine inside the DBD of hPPAR*γ* reduces PPAR*γ*-dependent transactivation (Figure 4). These cysteines are localized in two zinc finger motifs, each consisting of four cysteines complexing one zinc ion according to a NCBI conserved domain search. The oxidation of the thiols could therefore lead to a release of Zn^2+^ and a disruption of the zinc finger motif, resulting in a loss of DNA-binding activity [63]. Given that an oxidation of cysteines is probably cellular controlled by phosphorylation of hPPAR*γ*, this loss of the DNA-dependent regulator function could shift the function of the transcription factor to its DNA-independent role. Additionally, we analysed a hPPAR*γ* mutant of cysteine 284, which is located in the ligand-binding domain (LBD), and detected an altered interaction profile with ligands compared to the wt construct. C284 of the transcription factor is known for its connection to ligand-binding [64]. Therefore, a redox-modified C284 could change the ligand-binding profile of hPPAR*γ*. Although the existence of a redox PTM at C284 could not be validated via MS due to missing protein coverage, the observation of hPPAR*γ* C284A transactivator activity is in line with other established studies of phosphorylated S82 of the transcription factor, which reported reducing ligand binding affinity of the protein [52]. One limitation of our transactivator mutation studies is that we cannot completely exclude that the observations made are based exclusively on redox regulations but on conformational changes as well. Hence, we hypothesize, that redox-modified hPPAR*γ* features its main functionality in regulating processes in a DNA-independent way probably by affecting on differential protein-protein interactions. In this regard, it is already known that nuclear PPAR*γ* can regulate transcription also exclusively by its interaction with corepressors and coactivators without binding to DNA [65]. Furthermore, in the cytosol, the protein can interact directly with kinases, i.e., MEK1 and PKC*α*, and cytochrome c reductase [66–69]. The advantage of such DNA-independent mechanisms is enrooted at the basis of a relative fast initiation of desired cellular effects, whereas a DNA-dependent mode of action can take hours due to their genomic processing structure [70]. Therefore, for a fast establishment of a specific M*Φ* phenotype, the presence of a redox-modified PPAR*γ* could be beneficial.

Supporting the assumption of an altered interaction profile of redox-modified hPPAR*γ*, we observed that the protein-protein-interaction of hPPAR*γ* with the intermediate filament protein vimentin (VIM) and tubulin beta-5 chain (TUBB5) was altered upon redox stimulation of the macrophages. For both proteins, hPPAR*γ*-binding was maximal following LPS/IFN*γ*- and TPA-stimulation and minimal after IL4-treatment (Figure 6). VIM and TBB5 are important components of the cellular cytoskeleton, forming a filament network between nucleus, endoplasmatic reticulum (ER), and mitochondria [71, 72]. Therefore, their interaction with hPPAR*γ* could suggest a specific cellular transport mechanism of redox altered PPAR*γ* through the cytoplasm probably to the Golgi, mitochondria, and ER, where a general interaction of VIM and PPAR*γ* has already been noticed [73]. Therefore, a possible connection of these phospho-sites to this task still needs to be proven. Additionally, accurate functions of redox-modified hPPAR*γ*, which is characterized by the aspects of phosphorylation and cysteine oxidation, and its conformation, are still unknown and need to be further addressed in future research.

## 5. Conclusion

**Figure 8:**
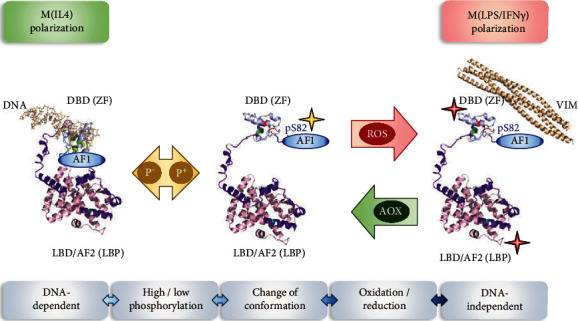
Overview of polarization-initiated changes of hPPAR*γ* due to S82 phosphorylation and redox-based PTMs. M(LPS/IFN*γ*) causes a phosphorylation (p^+^) at the N-terminal AF1 domain of hPPAR*γ* at serine 82, which results in a change of conformation of the protein. This makes the cysteines inside the zinc finger motif (ZF) of the DBD domain and probably also in the ligand-binding pocket (LBP) of the LBD/AF2 domain accessible for cellular ROS, allowing their oxidation. This in turn causes a change of hPPAR*γ* function from DNA-dependent to DNA-independent by promoting modified protein-protein interactions like the interaction of hPPAR*γ*-VIM. The shown hPPAR*γ* crystal structure during M(IL4) polarization is based on PDB ID: 3DZY with differential coloured functional domains of the protein [53]. The structure of a conformational changed protein was subsequently modified. Cysteine positions inside the DBD domain are highlighted in green (reduced state), respectively, red (oxidized state) and inside the LBP cyan (potentially oxidized, C284). The AF1 domain is due to its lack in the original crystal structure added afterwards. Stars mark the position of protein modifications (yellow≙phosphorylation, red≙oxidization). The structure of VIM is based on PDB ID: 5WHF [74].

Our study showed a novel mechanism of redox-based PTM regulating hPPAR*γ*, which is initiated by the differential cellular redox milieus of polarized M*Φ* into the classical LPS/IFN*γ* and alternative IL4-activated phenotype. Thus, our data suggest a connection between the phosphorylation of S82 and oxidation of cysteines of hPPAR*γ* protein (Figure 8). These redox-modifications could trigger a functional shift of hPPAR*γ* from a DNA-dependent to a DNA-independent function altering its protein-protein interaction profile.

## Figures and Tables

**Table 1:** hPPAR*γ* peptides after LysC digestion. Amino acids are stated in their single letter code. Peptide location inside of hPPAR*γ* is specified by amino acid (AA) position and their affiliation to the first and second zinc finger (ZF) and hinge domain (HD). Containing cysteines are noted.

hPPAR*γ* peptide	hPPAR*γ* position	Cysteine
TQLYNKPHEEPSNSLMAIECRVCGDK	AA 90-115 (1. ZF)	C109, C112
ASGFHYGVHACEGCK	AA 116-130 (1. ZF)	C126, C129
LIYDRCDLNCRIHK	AA 141-154 (2. ZF)	C146, C150
CQYCRFQK	AA 160-167 (2.ZF)	C160, C163
CLAVGMSHNAIRFGRMPQAEK	AA 168-188 (HD)	C168

## Data Availability

The data used to support the findings of this study are available from the corresponding author upon request.

## Abbreviations

AA: Amino acid
AF1/2: Transactivator domain 1 and 2
AOX: Antioxidants
DBD: DNA-binding domain
ER: Endoplasmatic reticulum
h: Human
HD: Hinge domain
IFN*γ*: Interferon gamma
IL4: Interleukin 4
LBD: Ligand binding domain
LBP: Ligand binding pocket
LPS: Lipopolysaccharide
m: Murine
M*Φ*: Macrophages
MAPK: Mitogen-activated protein kinase
MEK1: Mitogen-activated protein kinase kinase 1
MS: Mass spectrometry
NEM: N*-*ethylmaleimide
NOX2: NADPH oxidase 2 complex
PKC: Protein kinase C
PPAR*γ*: Peroxisome proliferator-activated receptor gamma
PTM: Posttranslational modification
RFI: Relative fluorescence intensities
RLU: Relative luminescence unit
roGFP2: Redox sensitive green fluorescent protein 2
ROS: Reactive oxygen species
RXR: Retinoid X receptor
pS82: Phosphorylation at serine 82 of PPAR*γ*
STAT6: Signal transducer and activator of transcription 6
TPA: Tetradecanoylphorbol acetate
Trx1: Thioredoxin 1
TUBB5: Tubulin *β*-5 chain
VIM: Vimentin
ZF: Zinc finger motif.

## Additional Points

*Highlights.* (i) Classical macrophage polarization by LPS/IFN*γ* causes an oxidizing, while IL4 activation a reductive intracellular redox milieu. (ii) Macrophage polarization causes redox-regulation of PPAR*γ*. (iii) Oxidation of PPAR*γ* is triggered by a nonredox-based stimulus. (iv) Redox-modification of PPAR*γ* alters DNA binding and protein-protein interaction

## Supplementary Materials

Supplementary Materials Methods for RNA extraction, qPCR, and the BIAM switch assay. Validation of the roGFP2 readout system and expression of polarization target genes (iNOS vs. Arginase1). Impact of short term redox stress on the redox status of cytosolic and nuclear proteins. Protein input of carried out LC/MS and representative MS spectra of PPAR*γ* peptides derived from AA positions 90-188. Heavy (D5-NEM) to light (NEM) ratios of hPPAR*γ* cysteines after redox stress and the validation of H_2_O_2_-induced PTMs at the cysteines of proteins via BIAM switch assay [75].
