# Supplementary material for: Redox Regulation of PPARγ in Polarized Macrophages
Source: PPAR Res. 2020 Jul 1;2020:8253831. doi: 10.1155/2020/8253831 (PMC7350077; doi:10.1155/2020/8253831)
Supplement: Supplementary Materials — Methods for RNA extraction, qPCR, and the BIAM switch assay. Validation of the roGFP2 readout system and expression of polarization target genes (iNOS vs. Arginase1). Impact of short term redox stress on the redox status of cytosolic and nuclear proteins. Protein input of carried out LC/MS and representative MS spectra of PPARγ peptides derived from AA positions 90-188. Heavy (D5-NEM) to light (NEM) ratios of hPPARγ cysteines after redox stress and the validation of H2O2-induced PTMs at the cysteines of proteins via BIAM switch assay [75]. [file 8253831.f1.pdf]

Supplementary Methods

1.1. RNA extraction and quantitative real-time PCR (qPCR)

Total RNA from cells was isolated using peqGOLD RNAPurereagent (PEQLAB Biotechnologie GmbH, Erlangen, Germany) and transcribed by utilizing Maxima first-strand cDNA synthesis kit (Thermo Scientific, Dreieich, Germany), each according to the manufacturer's protocol. qPCR assays were performed using IQ SYBR® Green Supermix (Bio-Rad Laboratories GmbH, Munich, Germany) on the CFX96 Touch™ Real-Time PCR (Bio-Rad) cycler. Data were analyzed via CFX Manager™ 3.1 software (Bio-Rad) and $\Delta\Delta C_t$ were calculated. 18S rRNA was used as internal control. Primer sequences are listed in Supplementary Tab. 1. Inducible nitric oxide synthase (*iNOS*) mRNA expression (Mm_LOC673161_1_SG) was determined using QuantiTect Primer Assay (QT01547980) from Qiagen (Hilden, Germany).

Supplementary Tab. 1 qPCR primers used in this study.

Target	Forward primer (5' - 3')	Reverse Primer (5' - 3')
18S	GTAACCCGTTGAACCCCATT	CCATCCAATCGGTAGTAGCG
Arg1	GTGAAGAACCCACGGTCTGT	CTGGTTGTCAGGGGAGTGTT

1.2. BIAM switch assay

Cellular available reduced cysteines of total protein content of HEK293T stably expressing HA-tagged hPPARγ were labeled by NEM (20 mM in DMEM) for 10 min on ice, before cells were washed 3 times with PBS and harvested afterwards. Further sample preparation started with cell lyses, followed by labeling of cellular oxidized cysteines by biotin-iodacetamide (BIAM) and finally biotin-immunoprecipitation as described elsewhere[75]. Samples were analyzed by Western blot studies, using detection of peroxiredoxin 3 (Prx3) as control for cellular redox stress.

Supplementary Figures

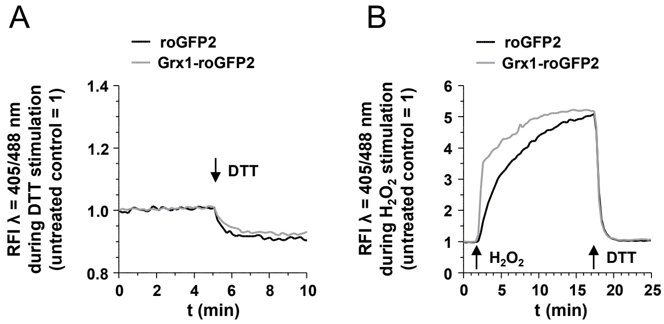

Supplementary Fig. 1. Validation of roGFP2 readout system to determine the redox status of MΦ. J774A.1 cells were transduced with vectors encoding the redox sensitive marker proteins roGFP2 or Grx1-roGFP2. To standardize the experimental setup, the fluorescence of untreated cells was initially monitored at 405 nm and 488 nm. To inflict changes in cellular redox milieu, cells were treated either by 10 mM DTT to cause reducing **(A)** or 10 mM H_2O_2 to induce oxidizing conditions **(B)**. Fluorometric changes were followed till a constant value was reached. Untreated cells were set as 1.

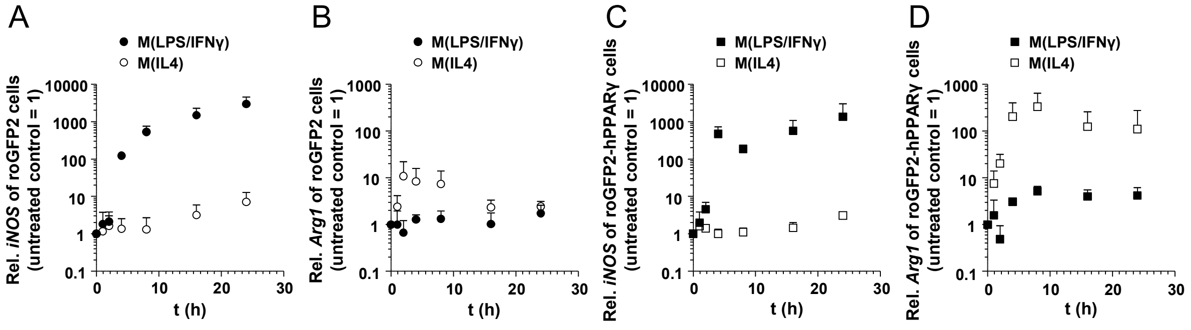

Supplementary Fig. 2. Effective macrophage polarization in response to LPS/IFNγ and IL4 was verified by expression of corresponding target genes. J774A.1 cells were stably transduced with vectors encoding the redox sensitive marker proteins roGFP2 **(A, B)** or roGFP2-hPPARγ **(C, D)**. Cells were harvested at the indicated time points. mRNA expression of target genes was normalized to 18S rRNA. Mean values ± SD are provided. Untreated control cells were set as 1.

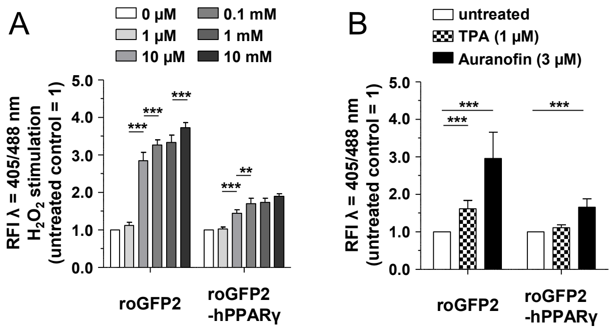

Supplementary Fig. 3. Impact of short-term redox stress on redox status of cytosolic and nuclear proteins. Lentiviral transduced J774A.1 MΦ expression cytosolic roGFP2 and nuclear roGFP2-hPPARγ were treated with the different redox stress inducers H_2O_2 (1 µM – 10 mM, 15 min) **(A)**, auranofin (3 µM, 40 min) and TPA (1 µM, 30 min) **(B)**. The effect of the stimulation was monitored by FACS-analysis at 405 nm and 488 nm. Quantification (Mean values ± SD) of flow cytometric data is provided in **(A, B)**. All experiments were performed at least three times. Untreated control cells were set as 1. (**p ≤ 0.01, ***p ≤ 0.001).

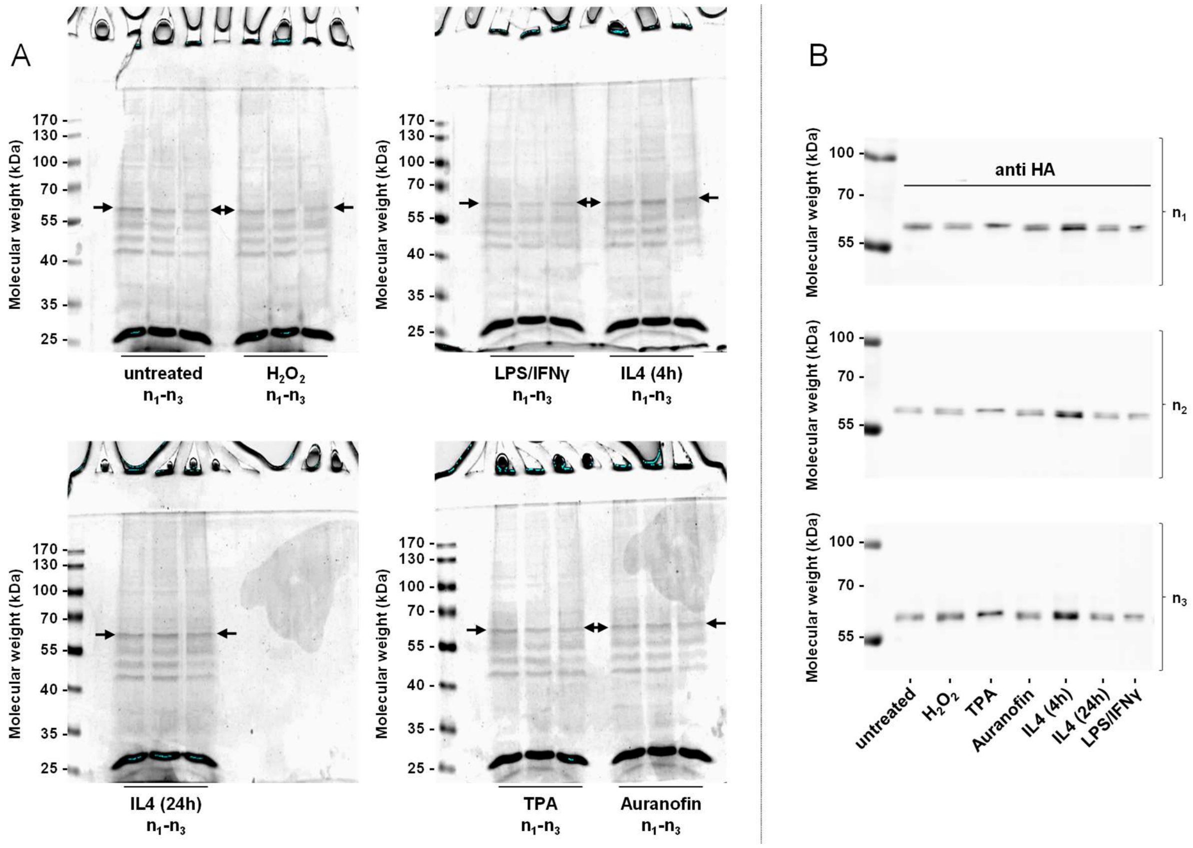

Supplementary Fig. 4. Protein input of carried out LC/MS. J774A.1 cells were transduced using lentiviral particles with integrated N- and C-terminal HA-labelled hPPARγ. To induce redox stress, MΦ were treated by 1 µg/ml LPS combined with 10 U/ml IFNγ for 4 h, 10 ng/ml IL4 for 4 h and 24 h, H_2O_2 (100 µM, 15 min), auranofin (3 µM, 40 min), or TPA (1 µM, 30 min). Untreated cells were used as controls. The redox states of reduced cysteines were fixed by NEM before cell harvest on ice. HA-tagged hPPARγ was HA-immunoprecipitated and passed SDS-PAGE befor LC/MS. For this, three SDS gel pieces (n_1-n_3) containing HA-tagged hPPARγ of each sample treatment were separately cut out of the coomassie stained SDS gel at the indicated areas and later on used as LC/MS samples **(A**, arrows**)**. Western blot studies were performed by using an anti HA antibody to determine the specific size of HA-tagged hPPARγ **(B)**.

A

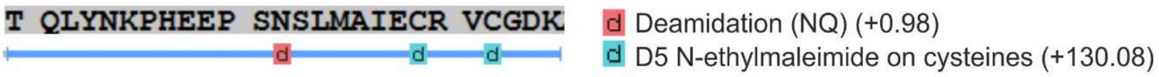

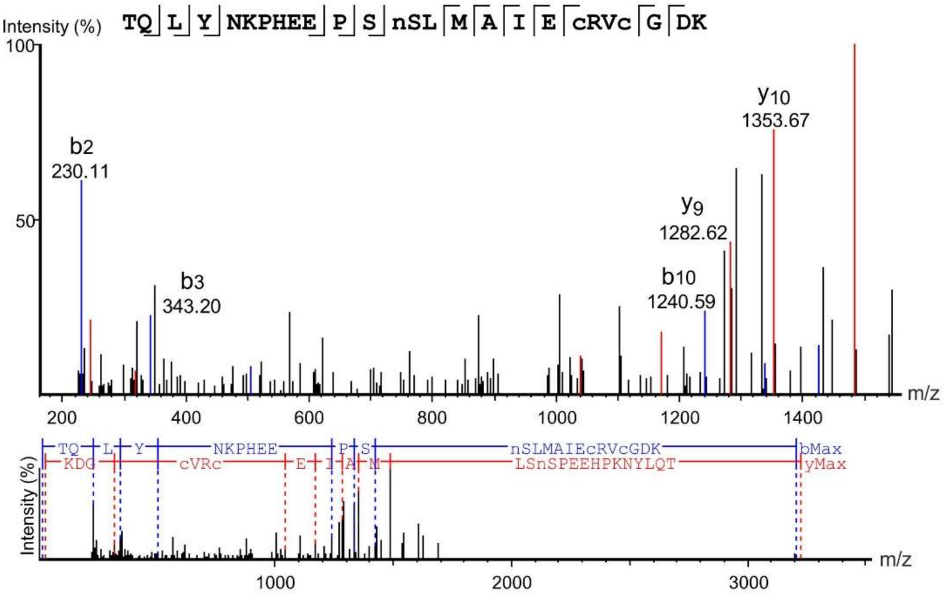

#	Immonium	b	b-H2O	Seq	y	y-H2O	#
1	74.06	102.06	84.04	T			26
2	101.07	230.11	212.10	Q	3122.48	3104.47	25
3	86.10	343.20	325.19	L	2994.42	2976.41	24
4	136.08	506.26	488.25	Y	2881.34	2863.33	23
5	87.06	620.30	602.29	N	2718.27	2700.26	22
6	101.11	748.40	730.39	K	2604.23	2586.22	21
7	70.07	845.45	827.44	P	2476.14	2458.13	20
8	110.07	982.51	964.50	H	2379.08	2361.07	19
9	102.06	1111.55	1093.54	E	2242.02	2224.01	18
10	102.06	1240.59	1222.59	E	2112.98	2094.97	17
11	70.07	1337.64	1319.64	P	1983.94	1965.93	16
12	60.04	1424.66	1406.67	S	1886.89	1868.88	15
13	88.04	1539.71	1521.70	N(+.98)	1799.85	1781.84	14
14	60.04	1626.74	1608.73	S	1684.83	1666.82	13
15	86.10	1739.82	1721.81	L	1597.80	1579.79	12
16	104.05	1870.86	1852.85	M	1484.71	1466.70	11
17	44.05	1941.90	1923.89	A	1353.67	1335.66	10
18	86.10	2054.99	2036.98	I	1282.62	1264.62	9
19	102.06	2184.03	2166.02	E	1169.55	1151.54	8
20	206.10	2417.12	2399.11	C(+130.08)	1040.51	1022.50	7
21	129.11	2573.22	2555.21	R	807.42	789.41	6
22	72.08	2672.29	2654.28	V	651.32	633.31	5
23	206.10	2905.37	2887.36	C(+130.08)	552.25	534.24	4
24	30.03	2962.40	2944.39	G	319.16	301.15	3
25	88.04	3077.42	3059.41	D	262.14	244.09	2
26	101.11			K	147.11	129.10	1

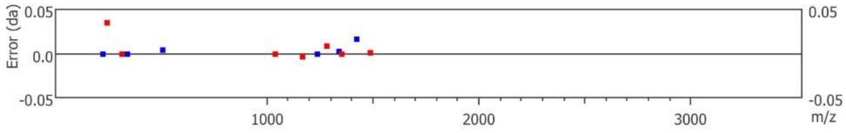

B

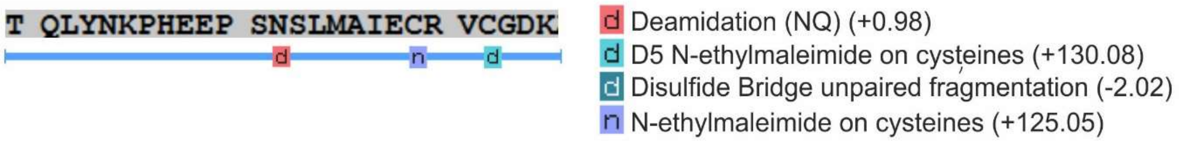

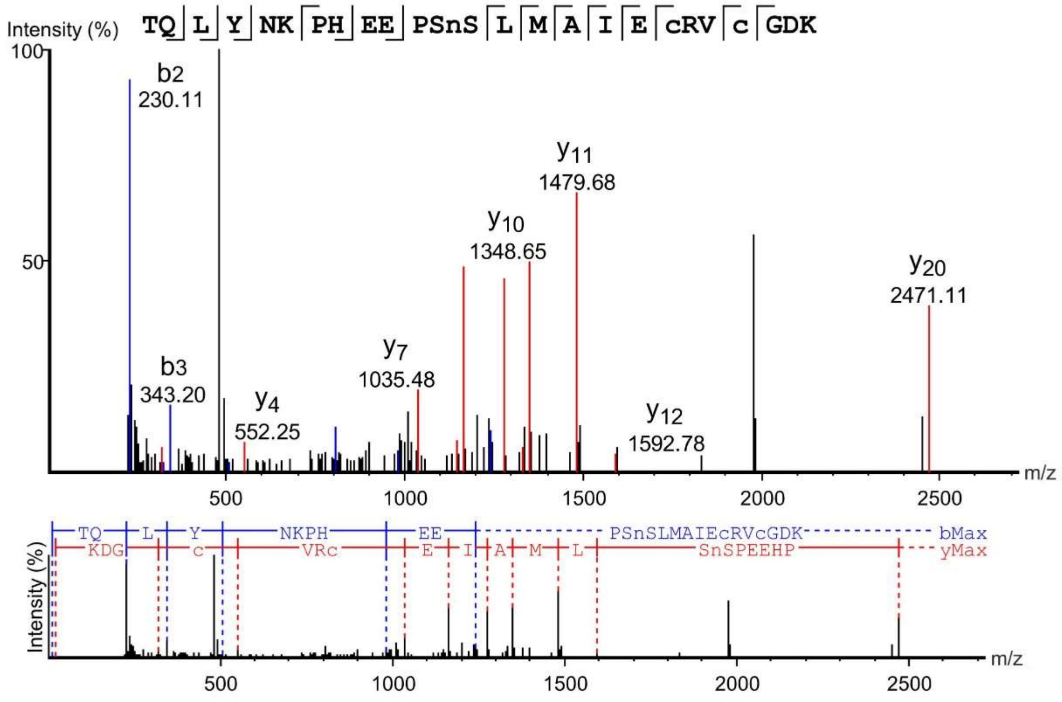

#	Immonium	b	b-H2O	Seq	y	y-H2O	#
1	74.06	102.06	84.04	T			26
2	101.07	230.11	212.10	Q	3117.45	3099.44	25
3	86.10	343.20	325.19	L	2989.39	2971.38	24
4	136.08	506.26	488.25	Y	2876.31	2858.30	23
5	87.06	620.30	602.29	N	2713.24	2695.23	22
6	101.11	748.40	730.39	K	2599.20	2581.19	21
7	70.07	845.45	827.44	P	2471.11	2453.09	20
8	110.07	982.49	964.50	H	2374.05	2356.04	19
9	102.06	1111.55	1093.54	E	2236.99	2218.98	18
10	102.06	1240.59	1222.59	E	2107.95	2089.94	17
11	70.07	1337.65	1319.64	P	1978.91	1960.90	16
12	60.04	1424.68	1406.67	S	1881.86	1863.84	15
13	88.04	1539.71	1521.70	N(+.98)	1794.82	1776.81	14
14	60.04	1626.74	1608.73	S	1679.80	1661.79	13
15	86.10	1739.82	1721.81	L	1592.78	1574.75	12
16	104.05	1870.86	1852.85	M	1479.68	1461.67	11
17	44.05	1941.90	1923.89	A	1348.65	1330.65	10
18	86.10	2054.99	2036.98	I	1277.61	1259.59	9
19	102.06	2184.03	2166.02	E	1164.51	1146.52	8
20	201.07	2412.09	2394.07	C(+125.05)	1035.48	1017.47	7
21	129.11	2568.19	2550.18	R	807.42	789.41	6
22	72.08	2667.25	2649.24	V	651.32	633.31	5
23	206.10	2900.34	2882.33	C(+130.08)	552.25	534.24	4
24	30.03	2957.36	2939.35	G	319.16	301.15	3
25	88.04	3072.39	3054.38	D	262.14	244.13	2
26	101.11			K	147.11	129.10	1

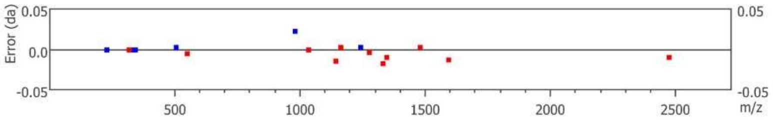

C

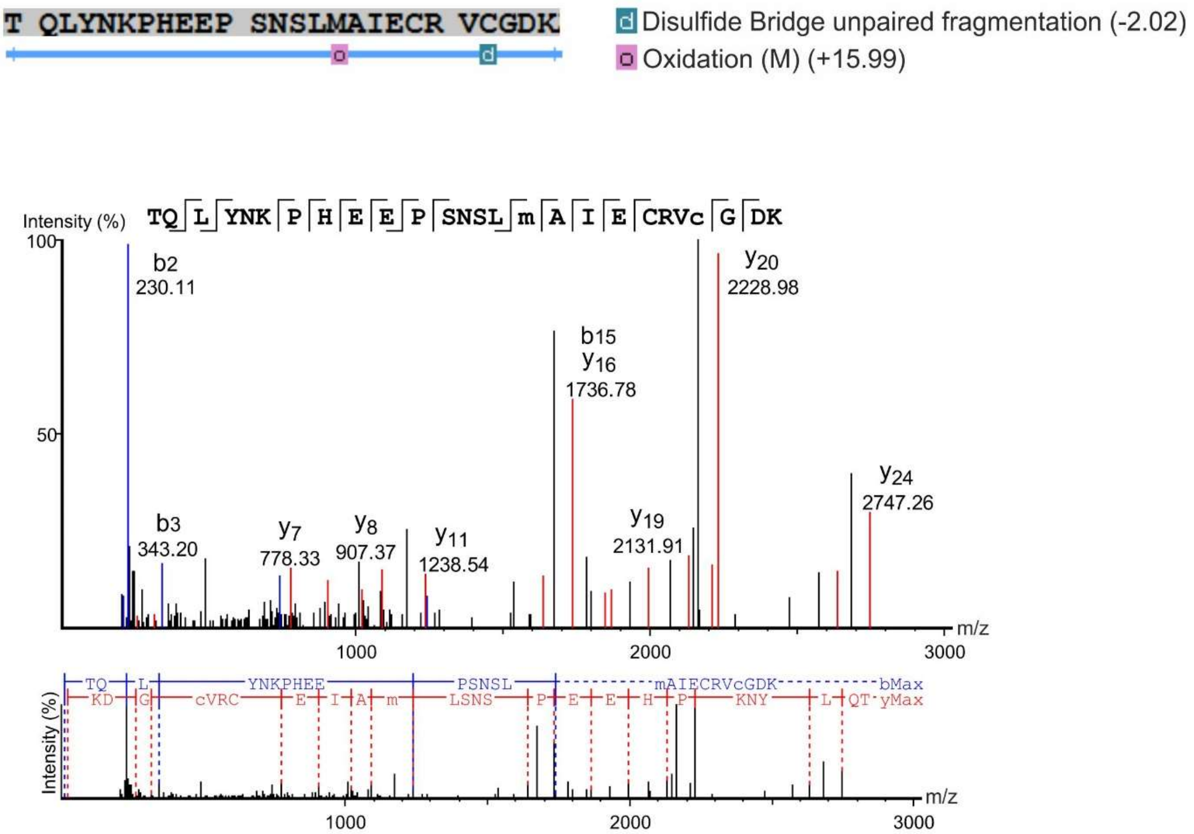

#	Immonium	b	b-H2O	Seq	y	y-H2O	#
1	74.06	102.06	84.04	T			26
2	101.07	230.11	212.10	Q	2875.31	2857.30	25
3	86.10	343.20	325.19	L	2747.26	2729.24	24
4	136.08	506.26	488.25	Y	2634.16	2616.16	23
5	87.06	620.30	602.29	N	2471.11	2453.10	22
6	101.11	748.40	730.39	K	2357.06	2339.05	21
7	70.07	845.45	827.44	P	2228.98	2210.97	20
8	110.07	982.51	964.50	H	2131.91	2113.91	19
9	102.06	1111.55	1093.54	E	1994.87	1976.85	18
10	102.06	1240.55	1222.59	E	1865.82	1847.82	17
11	70.07	1337.65	1319.64	P	1736.78	1718.76	16
12	60.04	1424.68	1406.67	S	1639.70	1621.71	15
13	87.06	1538.72	1520.71	N	1552.69	1534.68	14
14	60.04	1625.76	1607.75	S	1438.64	1420.63	13
15	86.10	1738.80	1720.83	L	1351.61	1333.60	12
16	120.05	1885.88	1867.86	M(+15.99)	1238.54	1220.52	11
17	44.05	1956.91	1938.90	A	1091.50	1073.48	10
18	86.10	2070.00	2051.99	I	1020.46	1002.45	9
19	102.06	2199.04	2181.03	E	907.37	889.36	8
20	76.02	2302.05	2284.04	C	778.33	760.32	7
21	129.11	2458.15	2440.14	R	675.32	657.31	6
22	72.08	2557.22	2539.21	V	519.22	501.21	5
23	74.00	2658.21	2640.20	C(-2.02)	420.15	402.14	4
24	30.03	2715.23	2697.22	G	319.16	301.15	3
25	88.04	2830.26	2812.24	D	262.16	244.13	2
26	101.11			K	147.11	129.10	1

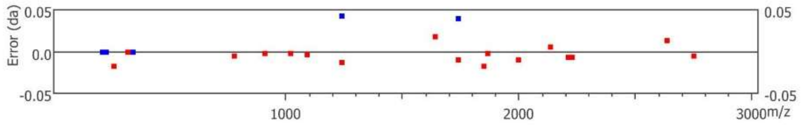

Supplementary Fig. 5. Representative MS2 spectra of a peptide at AA position 90-115. This peptide contains C109 and C112, shown in **(A)** with D5-NEM (m/z=645.5149, z=5, ppm=5.5, -10lgP=20.61), in **(B)** with D5-NEM, NEM, and disulfide (m/z=805.3776, z=4, ppm=-2.4, -10lgP=34.15), and in **(C)** with disulfide only (m/z=744.8454, z=4, ppm=-0.3, -10lgP=57.03). Ion tables shows fragment coverage by b- and y-ions.

A

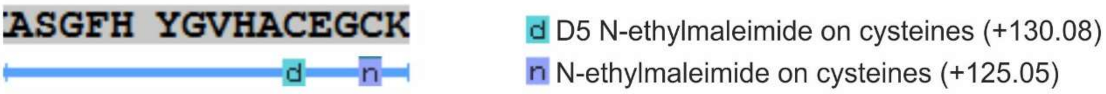

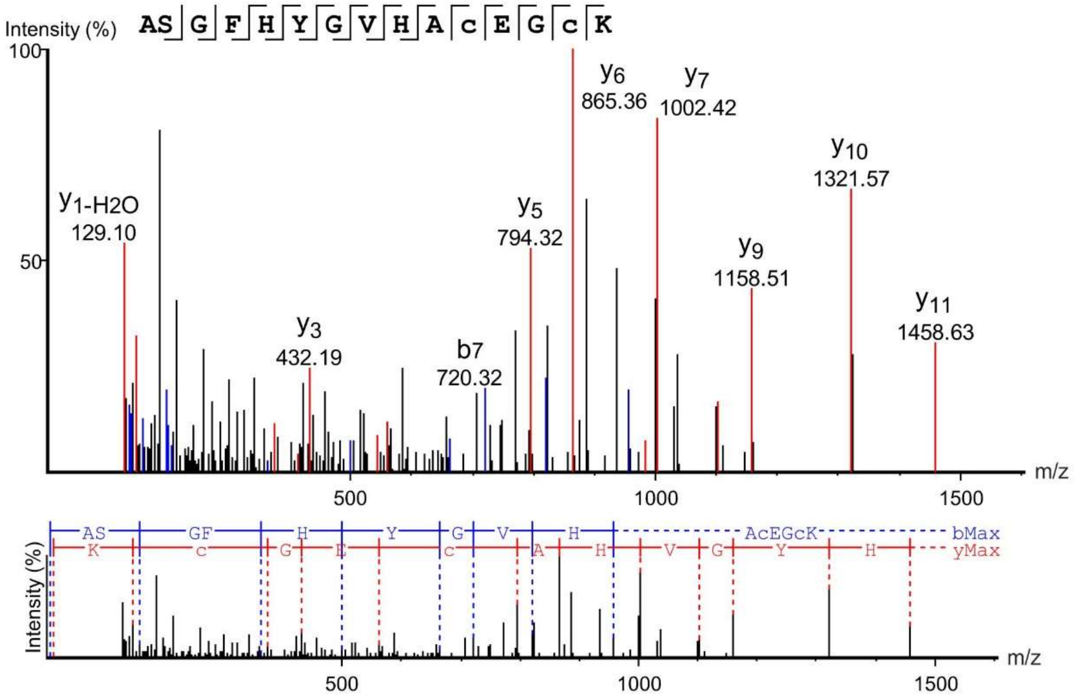

#	Immonium	b	b-H2O	Seq	y	y-H2O	#
1	44.05	72.04	54.03	A			15
2	60.04	159.08	141.07	S	1749.75	1731.74	14
3	30.03	216.10	198.09	G	1662.72	1644.71	13
4	120.08	363.14	345.16	F	1605.70	1587.69	12
5	110.07	500.22	482.22	H	1458.63	1440.62	11
6	136.08	663.30	645.28	Y	1321.57	1303.56	10
7	30.03	720.32	702.30	G	1158.51	1140.50	9
8	72.08	819.38	801.37	V	1101.48	1083.48	8
9	110.07	956.45	938.43	H	1002.42	984.41	7
10	44.05	1027.47	1009.46	A	865.36	847.35	6
11	206.10	1260.56	1242.55	C(+130.08)	794.32	776.31	5
12	102.06	1389.61	1371.60	E	561.23	543.23	4
13	30.03	1446.63	1428.62	G	432.19	414.20	3
14	201.07	1674.68	1656.67	C(+125.05)	375.17	357.16	2
15	101.11			K	147.11	129.10	1

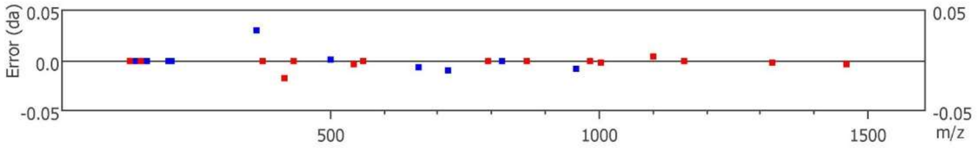

B

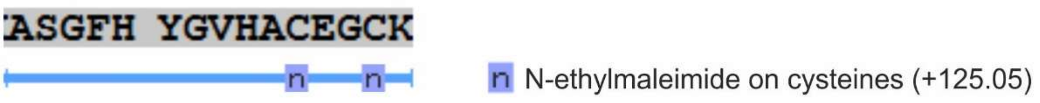

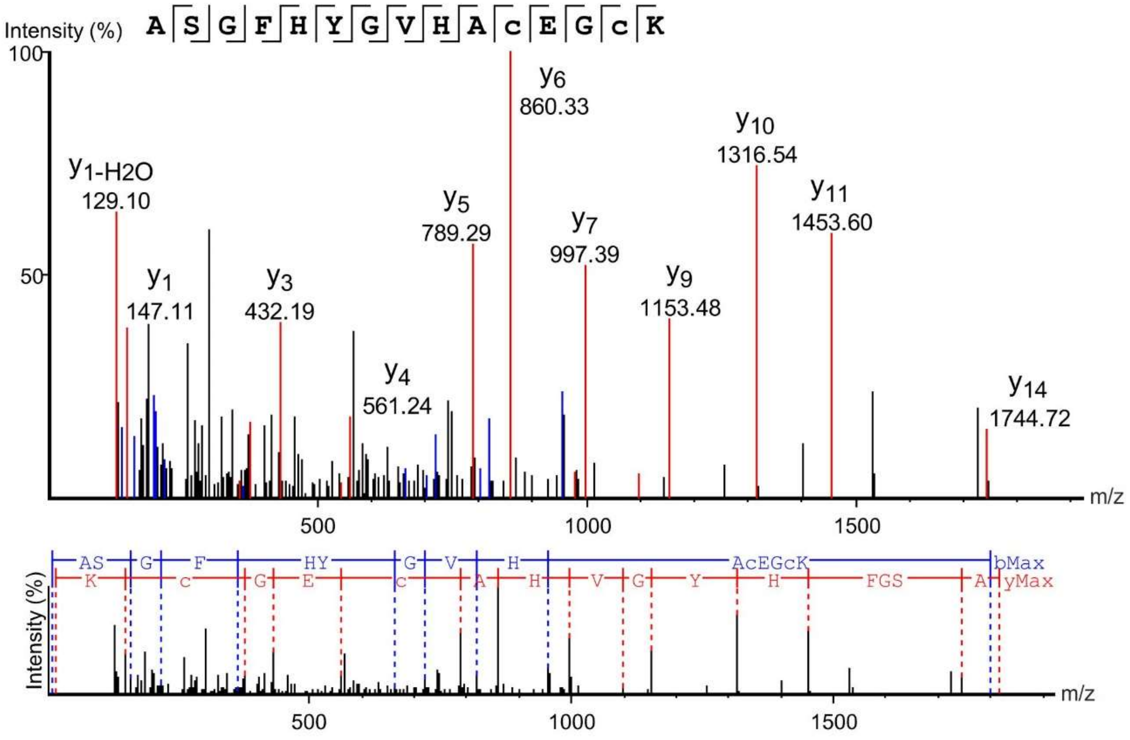

#	Immonium	b	b-H2O	Seq	y	y-H2O	#
1	44.05	72.04	54.03	A			15
2	60.04	159.08	141.07	S	1744.72	1726.71	14
3	30.03	216.10	198.09	G	1657.69	1639.68	13
4	120.08	363.18	345.16	F	1600.67	1582.66	12
5	110.07	500.23	482.22	H	1453.60	1435.59	11
6	136.08	663.29	645.28	Y	1316.54	1298.53	10
7	30.03	720.32	702.30	G	1153.48	1135.47	9
8	72.08	819.38	801.37	V	1096.46	1078.44	8
9	110.07	956.45	938.43	H	997.39	979.38	7
10	44.05	1027.47	1009.46	A	860.33	842.32	6
11	201.07	1255.53	1237.52	C(+125.05)	789.29	771.28	5
12	102.06	1384.57	1366.56	E	561.24	543.23	4
13	30.03	1441.60	1423.59	G	432.19	414.18	3
14	201.07	1669.65	1651.64	C(+125.05)	375.17	357.17	2
15	101.11			K	147.11	129.10	1

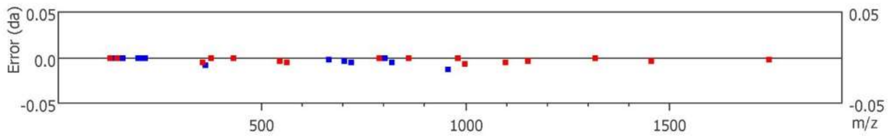

C

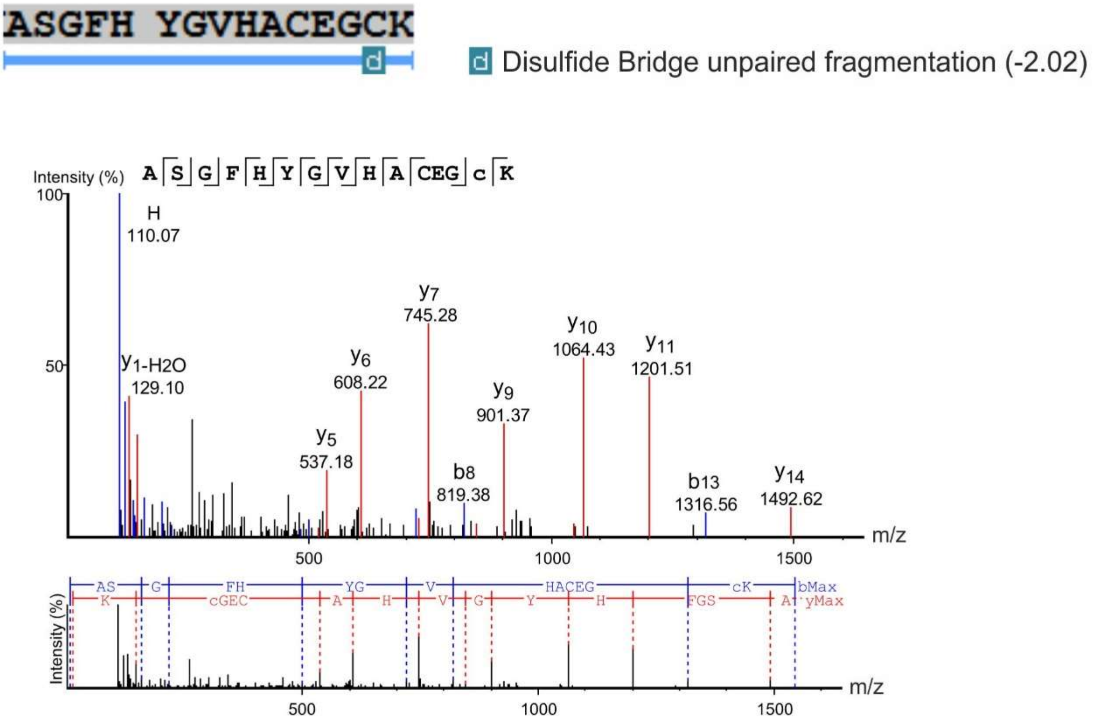

#	Immonium	b	b-H2O	Seq	y	y-H2O	#
1	44.05	72.04	54.03	A			15
2	60.04	159.08	141.07	S	1492.62	1474.59	14
3	30.03	216.10	198.09	G	1405.57	1387.56	13
4	120.08	363.17	345.16	F	1348.55	1330.54	12
5	110.07	500.23	482.21	H	1201.51	1183.47	11
6	136.08	663.29	645.28	Y	1064.43	1046.41	10
7	30.03	720.32	702.30	G	901.37	883.35	9
8	72.08	819.38	801.37	V	844.35	826.33	8
9	110.07	956.44	938.43	H	745.28	727.27	7
10	44.05	1027.47	1009.46	A	608.22	590.20	6
11	76.02	1130.48	1112.47	C	537.18	519.18	5
12	102.06	1259.53	1241.52	E	434.17	416.16	4
13	30.03	1316.56	1298.54	G	305.12	287.11	3
14	74.00	1417.54	1399.53	C(-2.02)	248.10	230.09	2
15	101.11			K	147.11	129.10	1

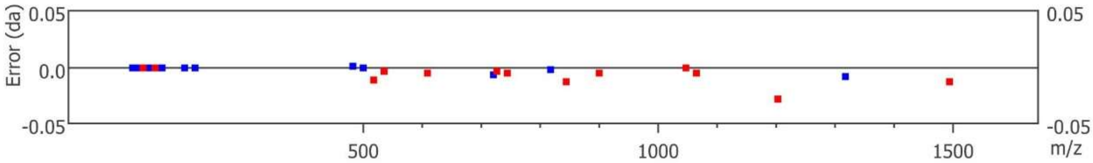

Supplementary Fig. 6. Representative MS2 spectra of a peptide at AA position 116-130. This peptide contains C126 and C129, shown in **(A)** with D5-NEM and NEM (m/z=607.6014, z=3, ppm=0.3, -10lgP=48.60), in **(B)** with NEM (m/z=605.9240, z=3, ppm=-0.3, -10lgP=59.65), and in **(C)** with a disulfide (m/z=521.8882, z=3, ppm=4.9, -10lgP=50.31). Ion tables shows fragment coverage by b- and y-ions.

A

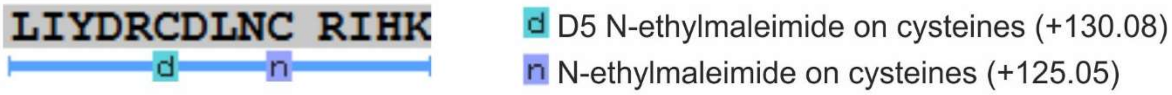

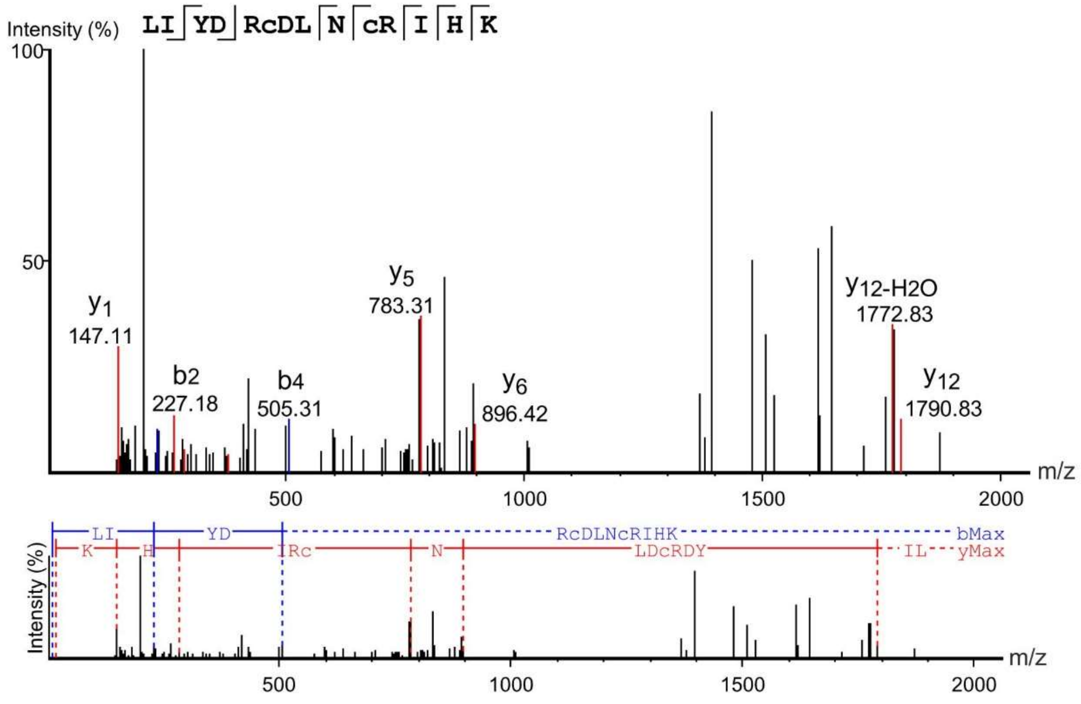

#	Immonium	b	b-H2O	Seq	y	y-H2O	#
1	86.10	114.09	96.08	L			14
2	86.10	227.18	209.17	I	1903.93	1885.92	13
3	136.08	390.24	372.23	Y	1790.83	1772.83	12
4	88.04	505.31	487.26	D	1627.78	1609.77	11
5	129.11	661.37	643.36	R	1512.76	1494.75	10
6	206.10	894.46	876.45	C(+130.08)	1356.66	1338.65	9
7	88.04	1009.48	991.47	D	1123.57	1105.56	8
8	86.10	1122.57	1104.56	L	1008.54	990.53	7
9	87.06	1236.61	1218.60	N	895.45	877.45	6
10	201.07	1464.67	1446.66	C(+125.05)	781.41	763.40	5
11	129.11	1620.77	1602.76	R	553.36	535.35	4
12	86.10	1733.85	1715.84	I	397.26	379.25	3
13	110.07	1870.91	1852.90	H	284.17	266.16	2
14	101.11			K	147.11	129.10	1

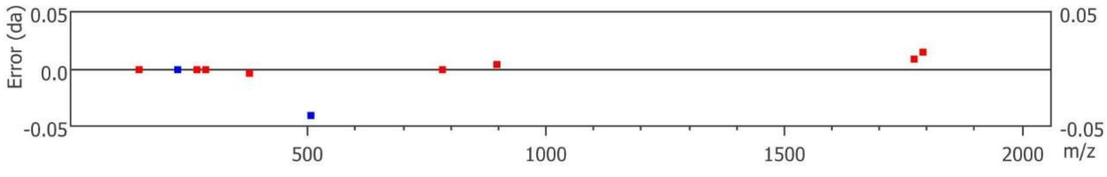

B

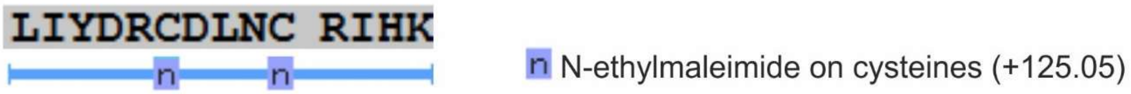

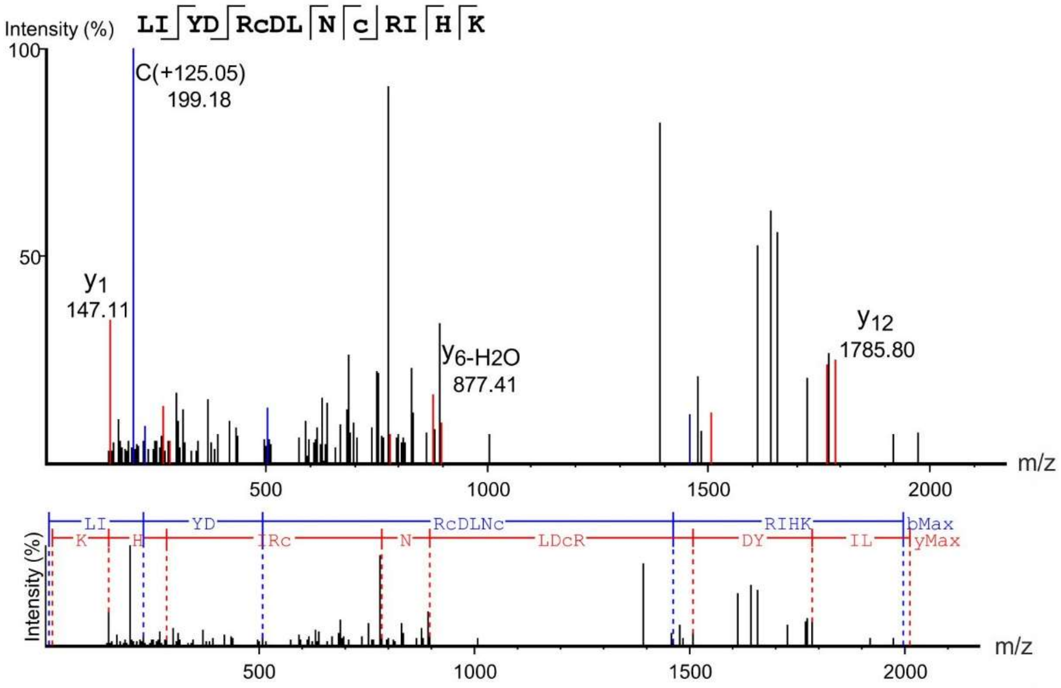

#	Immonium	b	b-H2O	Seq	y	y-H2O	#
1	86.10	114.09	96.08	L			14
2	86.10	227.18	209.17	I	1898.90	1880.89	13
3	136.08	390.24	372.23	Y	1785.80	1767.81	12
4	88.04	505.26	487.26	D	1622.75	1604.74	11
5	129.11	661.37	643.36	R	1507.70	1489.72	10
6	201.07	889.42	871.41	C(+125.05)	1351.62	1333.61	9
7	88.04	1004.45	986.44	D	1123.57	1105.56	8
8	86.10	1117.54	1099.52	L	1008.54	990.53	7
9	87.06	1231.58	1213.57	N	895.46	877.41	6
10	201.07	1459.62	1441.62	C(+125.05)	781.42	763.40	5
11	129.11	1615.74	1597.73	R	553.36	535.35	4
12	86.10	1728.82	1710.81	I	397.26	379.25	3
13	110.07	1865.88	1847.87	H	284.17	266.16	2
14	101.11			K	147.11	129.10	1

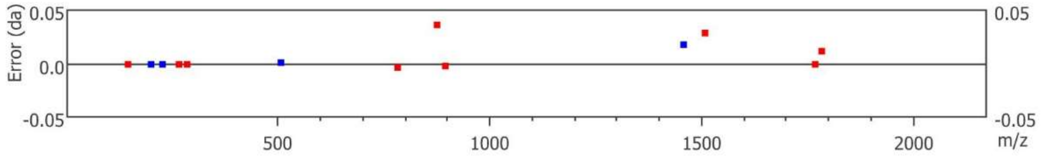

Supplementary Fig. 7. Representative MS2 spectra of a peptide at AA position 141-154. This peptide contains C146 and C150, shown in **(A)** with D5-NEM and NEM (m/z=505.0099, z=4, ppm=1.2, -10lgP=24.48), and in **(B)** with NEM (m/z=503.7509, z=3, ppm=-1.1, -10lgP=25.98). Ion tables shows fragment coverage by b- and y-ions.

A

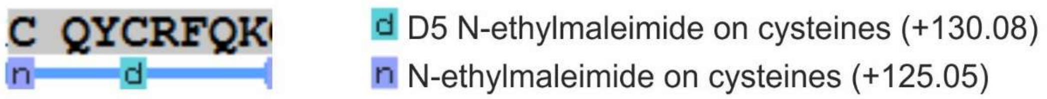

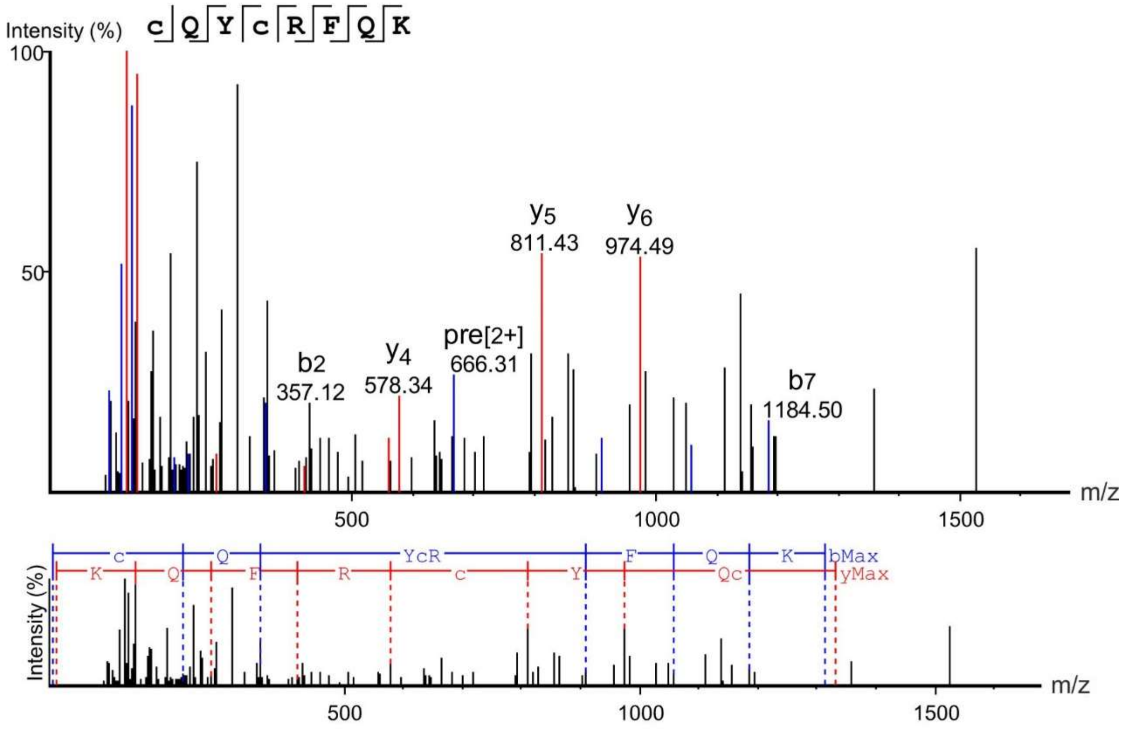

#	Immonium	b	b-H2O	Seq	y	y-H2O	#
1	201.07	229.06	211.05	C(+125.05)			8
2	101.07	357.12	339.11	Q	1102.55	1084.54	7
3	136.08	520.19	502.18	Y	974.49	956.48	6
4	206.10	753.27	735.26	C(+130.08)	811.43	793.42	5
5	129.10	909.37	891.37	R	578.34	560.34	4
6	120.08	1056.43	1038.43	F	422.24	404.23	3
7	101.07	1184.50	1166.49	Q	275.17	257.16	2
8	101.11			K	147.11	129.10	1

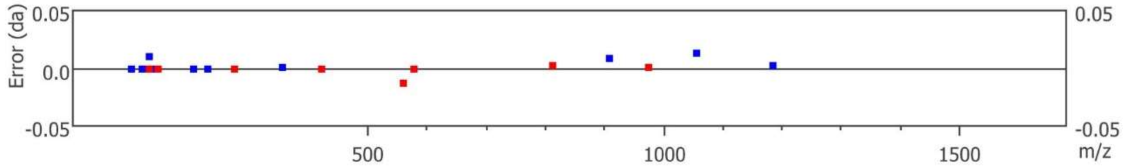

B

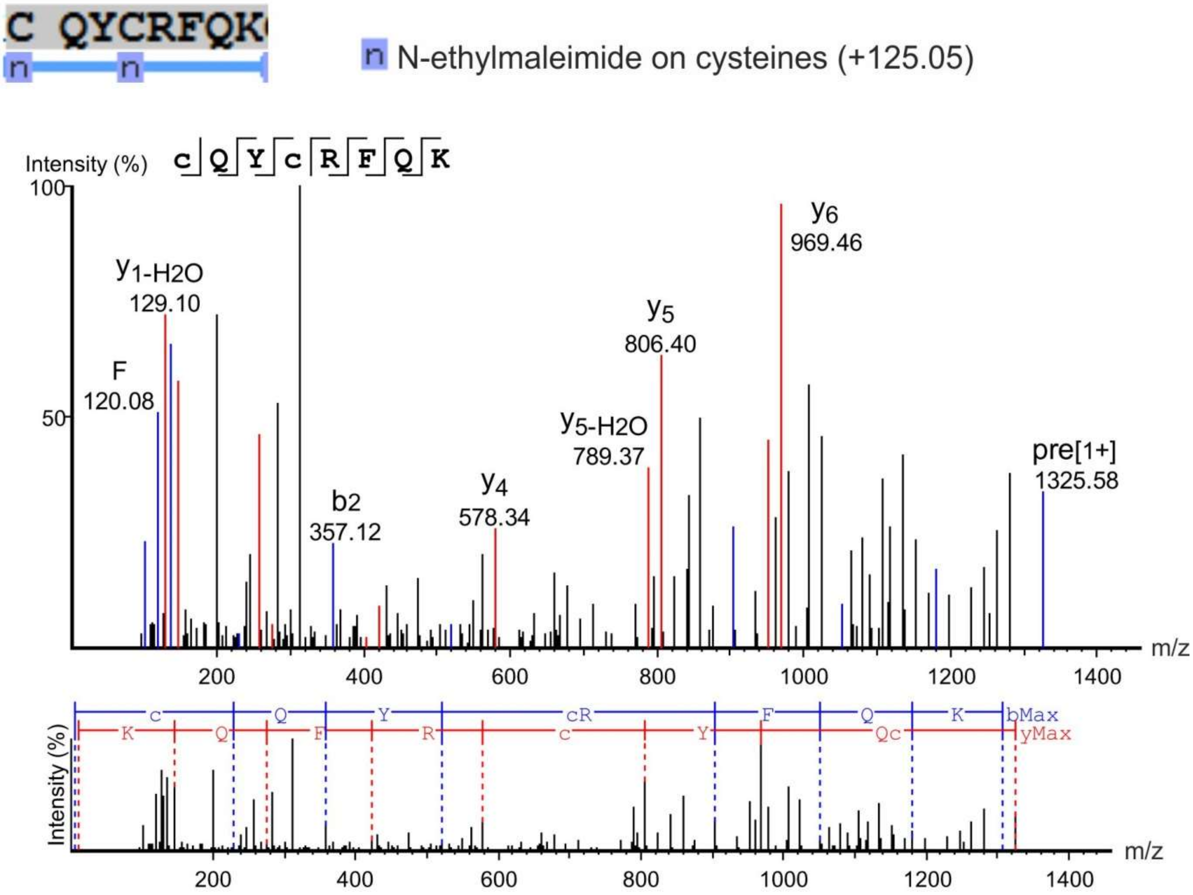

#	Immonium	b	b-H2O	Seq	y	y-H2O	#
1	201.07	229.06	211.05	C(+125.05)			8
2	101.07	357.12	339.11	Q	1097.52	1079.51	7
3	136.08	520.18	502.18	Y	969.46	951.44	6
4	201.07	748.24	730.23	C(+125.05)	806.40	788.38	5
5	129.10	904.34	886.33	R	578.34	560.33	4
6	120.08	1051.42	1033.40	F	422.24	404.24	3
7	101.07	1179.46	1161.46	Q	275.17	257.16	2
8	101.11			K	147.11	129.10	1

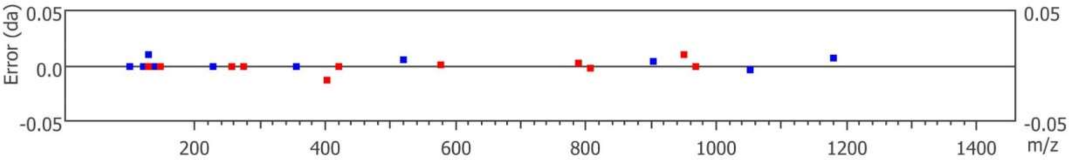

C

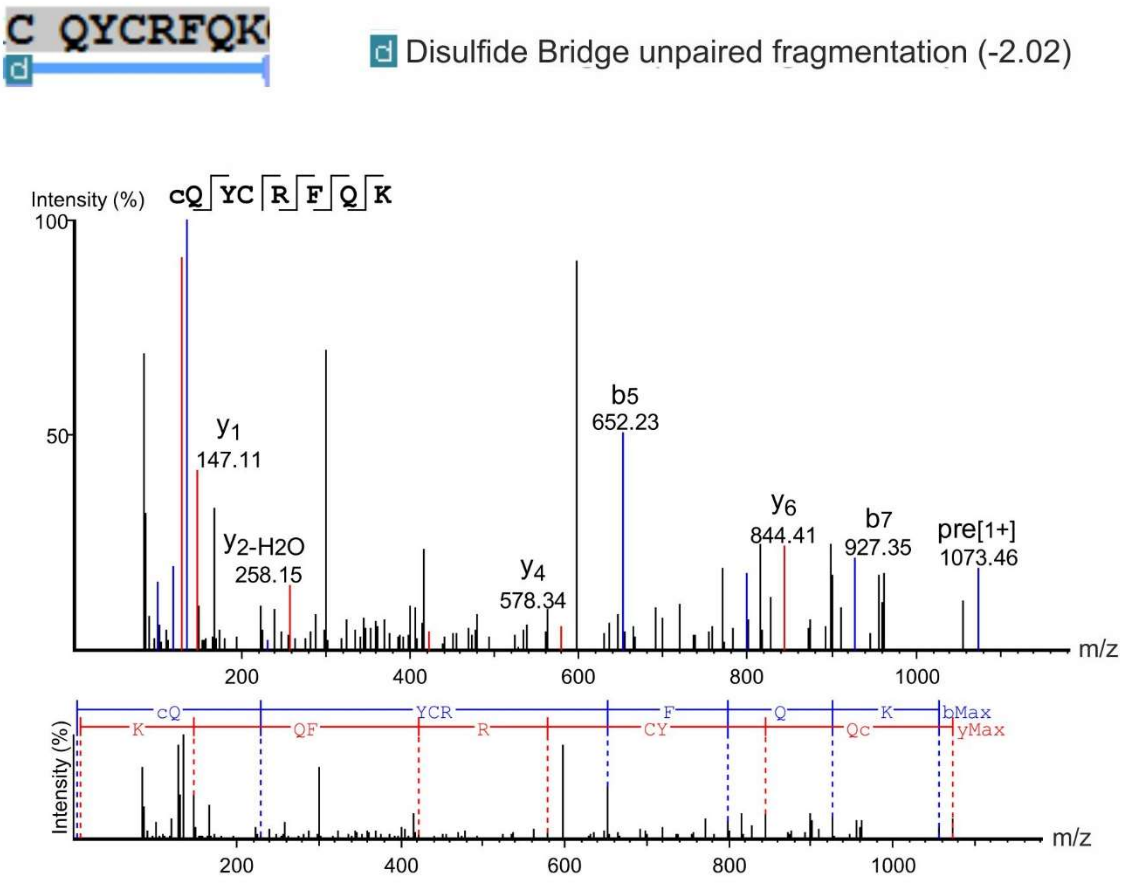

#	Immonium	b	b-H2O	Seq	y	y-H2O	#
1	74.00	102.00	83.99	C(-2.02)			8
2	101.07	230.06	212.05	Q	972.47	954.46	7
3	136.08	393.12	375.11	Y	844.41	826.40	6
4	76.02	496.13	478.12	C	681.35	663.34	5
5	129.10	652.23	634.22	R	578.34	560.33	4
6	120.08	799.29	781.29	F	422.24	404.23	3
7	101.07	927.35	909.35	Q	275.17	257.16	2
8	101.11			K	147.11	129.10	1

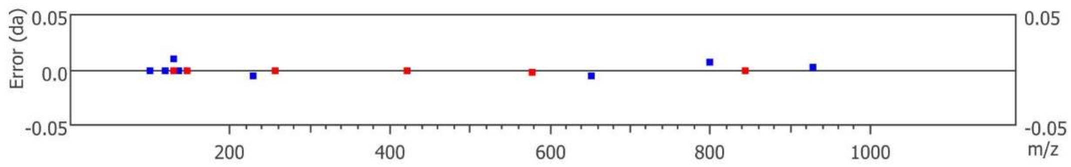

Supplementary Fig. 8. Representative MS2 spectra of a peptide at AA position 160-167. This peptide contains C160 and C163, shown in **(A)** with D5-NEM and NEM (m/z=665.8071, z=2, ppm=-0.7, -10lgP=27.36), in **(B)** with NEM (m/z=663.2920, z=2, ppm=0.2, -10lgP=37.87), and in **(C)** with a disulfide (m/z=537.2368, z=2, ppm=4.9, -10lgP=27.52). Ion tables shows fragment coverage by b- and y-ions.

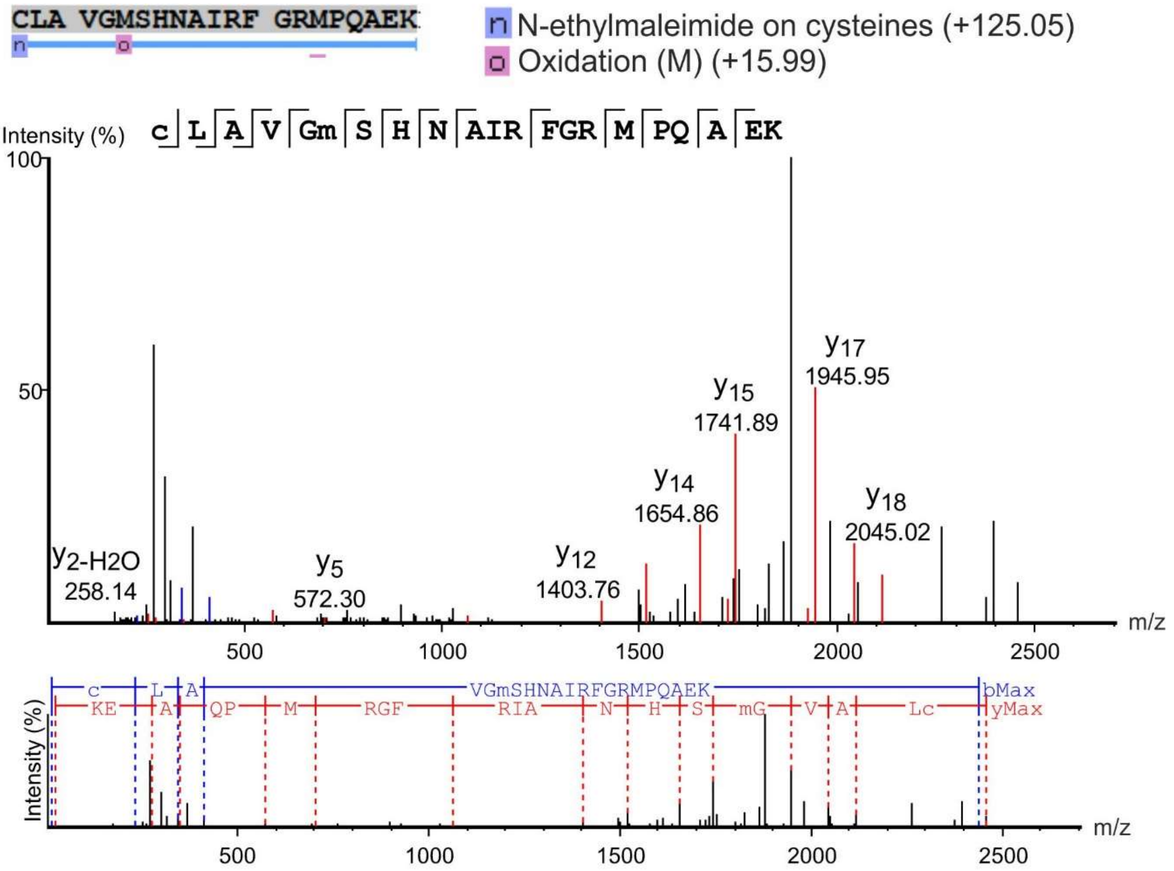

#	Immonium	b	b-H2O	Seq	y	y-H2O	#
1	201.07	229.06	211.05	C(+125.05)			21
2	86.10	342.15	324.14	L	2229.14	2211.13	20
3	44.05	413.19	395.18	A	2116.05	2098.04	19
4	72.08	512.25	494.24	V	2045.02	2027.01	18
5	30.03	569.28	551.27	G	1945.95	1927.93	17
6	120.05	716.31	698.30	M(+15.99)	1888.93	1870.92	16
7	60.04	803.34	785.33	S	1741.89	1723.88	15
8	110.07	940.40	922.39	H	1654.86	1636.85	14
9	87.06	1054.45	1036.43	N	1517.81	1499.79	13
10	44.05	1125.48	1107.47	A	1403.76	1385.75	12
11	86.10	1238.57	1220.56	I	1332.72	1314.71	11
12	129.11	1394.67	1376.66	R	1219.64	1201.63	10
13	120.08	1541.74	1523.73	F	1063.53	1045.52	9
14	30.03	1598.76	1580.75	G	916.47	898.46	8
15	129.11	1754.86	1736.85	R	859.45	841.43	7
16	104.05	1885.90	1867.89	M	703.37	685.33	6
17	70.07	1982.95	1964.94	P	572.30	554.29	5
18	101.07	2111.01	2093.00	Q	475.25	457.24	4
19	44.05	2182.05	2164.04	A	347.19	329.18	3
20	102.06	2311.09	2293.08	E	276.16	258.14	2
21	101.11			K	147.11	129.10	1

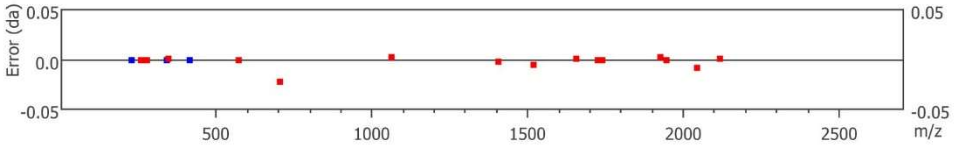

Supplementary Fig. 9. Representative MS2 spectra of a peptide at AA position 168-188. This peptide contains C168, shown with NEM (m/z=615.0542, z=0.1, ppm=0.2, -10lgP=48.30). Ion tables shows fragment coverage by b- and y-ions.

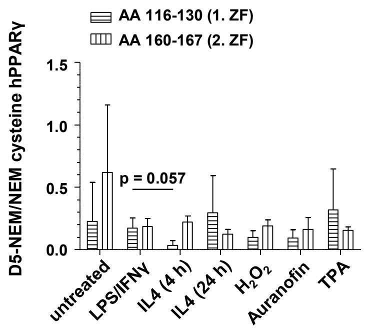

Supplementary Fig. 10. Heavy (D5-NEM) to light (NEM) ratios of hPPARγ cysteines after redox stress. J774A.1 cells were transduced using lentiviral particles with integrated N- and C-terminal HA-labelled hPPARγ. To induce redox stress, MΦ were treated by 1 µg/ml LPS combined with 10 U/ml IFNγ for 4 h, 10 ng/ml IL4 for 4 h and 24 h, H_2O_2 (100 µM, 15 min), auranofin (3 µM, 40 min), or TPA (1 µM, 30 min). Untreated cells were used as controls. The redox states of reduced cysteines were fixed by NEM before cell harvest on ice. HA-tagged hPPARγ was HA-immunoprecipitated, existing oxidized cysteines denatured and labelled by D5-NEM. Peptides were analysed by LC/MS after LysC digestion. Quantification of the ratio of cellular oxidized (D5-NEM) and reduced (NEM) cysteine are mapped. Peptides of different hPPARγ amino acid sequences (AA) are depicted in Table 1. Cysteines affiliations to the first and second zinc finger (ZF) are indicated. All experiments were performed at least three times. Mean values ± SD are provided.

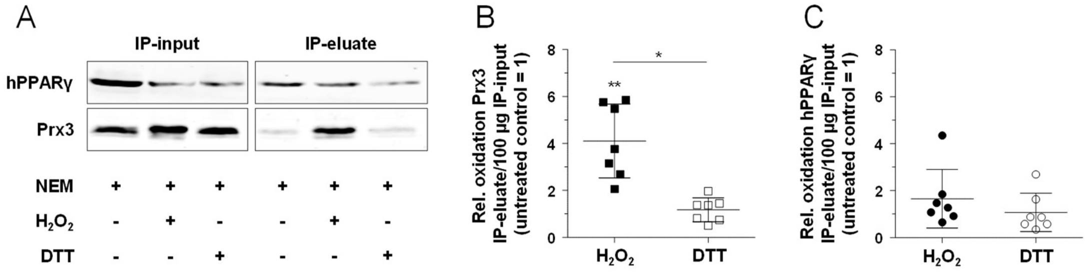

Supplementary Fig. 11. Validation of H_2O_2 induced PTMs at the cysteines of proteins via BIAM switch assay. HEK293T cells were transduced using lentiviral particles with integrated N- and C-terminal HA-labelled hPPARγ. To induce redox stress, cells were treated by 100 µM H_2O_2 as oxidizing or 100 µM DTT as reducing condition. Untreated cells were used as controls. The redox states of present reduced cysteines were fixed by NEM before cell harvest on ice. Remaining oxidized cysteines were reduced with 2.5 mM DTT, respectively, biotin labelled after cell lysis and protein-TCA precipitation. Immunoprecipitation was carried out against biotin-labelled cysteines (oxidized cysteines) and samples were analysed via Western blot. **(A)** shows a representative Western blot with total lysate (IP-input, each lane 50 µg) as loading control and the biotinylated IP-eluate. Positive induced redox stress was verified with protein intensities of Prx3 as control protein on each blot in parallel to hPPARγ. Quantification with mean values ± SD out of 7 separate experiments of Prx3 **(B)** and hPPARγ **(C)** is provided. Untreated control cells were set as 1 (* ≙ $p \leq 0.05$, ** ≙ $p \leq 0.01$).
